# Identification of Unknown Inverted Singlet–Triplet Cores by High-Throughput Virtual Screening

**DOI:** 10.1021/jacs.3c05452

**Published:** 2023-08-28

**Authors:** Ömer H. Omar, Xiaoyu Xie, Alessandro Troisi, Daniele Padula

**Affiliations:** † Department of Chemistry, University of Liverpool, Liverpool L69 7ZD, U.K.; ‡ Dipartimento di Biotecnologie, Chimica e Farmacia, Università di Siena, Via A. Moro 2, Siena 53100, Italy

## Abstract

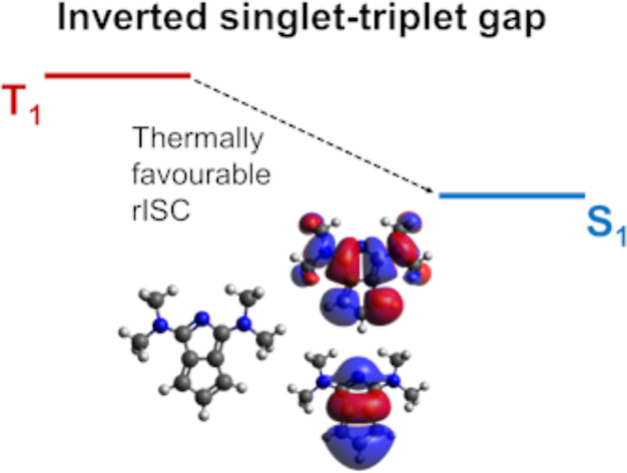

Molecules where the energy of the lowest excited singlet state is found below the energy of the lowest triplet state (inverted singlet–triplet molecules) are extremely rare. It is particularly challenging to discover new ones through virtual screening because the required wavefunction-based methods are expensive and unsuitable for high-throughput calculations. Here, we devised a virtual screening approach where the molecules to be considered with advanced methods are pre-selected with increasingly more sophisticated filters that include the evaluation of the HOMO-LUMO exchange integral and approximate CASSCF calculations. A final set of 7 candidates (0.05% of the initial 15 000) were verified to possess inversion between singlet and triplet states with state-of-the-art multireference methods (MS-CASPT2). One of them is deemed of particular interest because it is unrelated to other proposals made in the literature.

## 1 Introduction

Organic Light Emitting Diodes (OLEDs) find wide application in everyday life in lighting devices and consumer displays.^1^ OLED development’s major challenge is to increase their external quantum efficiency (EQE) to obtain brighter devices with lower power consumption.^2^ First generation OLEDs exploited fluorescence^3^ and their efficiency was limited by spin statistics of charge recombination, which only produces a small fraction (25%) of (emissive) singlet excitons.^4^ The initial strategy adopted to increase efficiency (2nd generation OLEDs) exploited phosphorescence, i.e. radiation emitted by the 75% of excited states formed by charge recombination in the triplet state.^5^ Since 2012, the 3rd generation of OLEDs exploit dyes able to undergo thermally activated delayed fluorescence (TADF),^6,7^ because it can provide high efficiency materials without the need for expensive heavy elements. TADF can take place when the energy gap between the lowest excited singlet and triplet states (Δ*E*_ST_) is comparable with thermal energy. The emission from the singlet is initiated by a process of reverse intersystem crossing (rISC) from the triplet^8^ and OLEDs based on this design have shown EQEs up to ≈40%.^9^ The current strategy to design TADF dyes relies on achieving a small singlet–triplet gap,^10−12^ obtained through a spatial separation of the highest occupied molecular orbital (HOMO) and least unoccupied molecular orbital (LUMO), often achieved in donor–acceptor architectures.^13,14^ The highly uncommon situation where the first excited singlet is energetically below the lowest triplet state (Δ*E*_ST_ < 0)^15^ is known as an inverted singlet–triplet (also as IST or INVEST in literature) and is a violation of Hund’s rule.^16^ This inversion is deemed beneficial for applications^17^ because it speeds up rISC, responsible for populating the (singlet) emissive state from the (triplet) harvesting state; as a matter of fact, the next-generation improvement of TADF dyes is the design of materials where rISC is fast.^18^ Early examples of this successful strategy appeared from 2013, when Adachi reported an increased EQE from ≈5% to ≈18%.^19,20^ The initial interpretation assumed a small positive singlet–triplet gap, but it was recently revised in light of singlet–triplet inversion.^21^

Singlet–triplet inversion originates from the interplay between the exchange interaction, a positive term promoting the stabilization of the triplet with respect to the singlet, and electron correlation, arising from the presence of doubly excited configurations (and/or higher order excitations), which may introduce a stabilization of the singlet with respect to the triplet.^15^ In a simple two-state model (i.e., a HOMO → LUMO transition), the exchange integral *K*_HL_ between HOMO and LUMO determines the splitting as Δ*E*_ST_ = 2*K*_HL_^15,22^ and it is always positive. The singlet–triplet inversion is achieved by the interaction with at least double excitations that can only be studied using wavefunction based methods,^15^ or at least double-hybrid density functionals.^16^ In fact, if it is clear that singlet–triplet inversion is attributable to electronic correlation, it is not straightforward to discern whether static (due to the multireference character of the wavefunction) or dynamic correlation has a dominant effect.

Not many chromophores where singlet–triplet inversion occurs are known; current literature efforts are essentially limited to triangulenes and their derivatives.^23−27^ Additionally, the very feature of singlet–triplet inversion is due to a significant double excitation character of the singlet state,^15^ which also leads to low oscillator strengths and, thus, dim fluorescence, in clear contrast with the final application domain. Design rules to modify triangulene cores to achieve IST gaps with acceptable oscillator strengths have been recently provided,^28^ but the limitation of the narrow range of available starting materials still remains. Virtual screening for IST materials could offer a solution presenting challenges that are not found in other virtual screening campaigns. Relatively inexpensive time dependent-density functional theory (TD-DFT) calculations are not sufficiently accurate for this problem^15^ and can only be used to preselect the candidates to be screened. The only possible approach to increase the degree of confidence in the screening process is to perform high-level calculations on the best candidates. Pollice et al. reported a screening on IST materials, studying a large set of nitrogen-substituted triangulenes.^26^ Aizawa et al. screened ≈35 K heptazine derivatives, a specific nitrogen-substituted triangulene core, identifying candidates that were also experimentally confirmed.^23^ A set of new extended-triangulene cores were recently proposed by Ricci et al. in their quest to outline design rules for IST materials,^28^ and these new cores could result in further similar screenings. However, since the triangulene and heptazine cores are already known to provide IST materials, these screenings were essentially aimed at identifying compounds with appreciable oscillator strength.^28^ Nevertheless, to identify further design rules to achieve IST, one needs to discover other cores displaying such feature, something that cannot be achieved if the chemical space where the search is performed is defined by the known examples. New cores with unusual properties can be identified *via* virtual screening of datasets of organic compounds not originally designed for a particular function.^29,30^ This “repurposing” approach was adopted by us to identify several families of new lead compounds to be used as singlet fission materials,^31^ TADF emitters,^32^ non-fullerene acceptors,^33^ and charge and exciton transport materials.^34,35^ An analogous step in the direction of IST candidates was recently taken by Blaskovits et al.;^36^ screening a set of known organic molecules, they identified two new classes of IST molecules, one of which seems particularly promising for applications in OLEDs, and where the singlet–triplet inversion mechanism is driven by high symmetry and stabilization through aromaticity.

In this work, we computationally search for new IST lead cores in a wider chemical space, starting from the data sets we presented in previous works.^37^ In the set of identified molecules, we provide a completely new core that can, thus, provide new avenues to the 3rd generation of TADF-based OLEDs.

## 2 Materials and Methods

We summarize the steps for this screening as follows (details of each step are given below):

1. A set of 11 molecules known to exhibit IST are evaluated with a range of methods proposed in the literature, including inexpensive proxy properties that can be used to preselect the best candidates such as TDDFT calculations and *K*_HL_; by known IST molecules we mean molecules for which the consensus across different computational methods has shown Δ*E*_ST_ ≤ 0, since experimental confirmation is still challenging owing to the dark nature of the states involved;
2. A subset of ≈15 000 molecules from existing larger databases of TDDFT calculations is extracted on the basis of Δ*E*_ST_^TDDFT^ calculations, with cutoff derived from step 1;
3. The molecules considered are reduced to 760 on the basis of rotatable bonds, *K*_HL_ (again suggested by analysis in step 1) and lack of CT character;
4. Δ*E*_ST_^CASSCF^ was computed at both CASSCF(6,6) and CASSCF(8,8) on 760 residual molecules and for 7 molecules, one of the 2 levels predicts Δ*E*_ST_^CASSCF^ ≤ 0;
5. Higher-level calculations are performed for the 7 candidates to further confirm IST.

**Figure 1:**
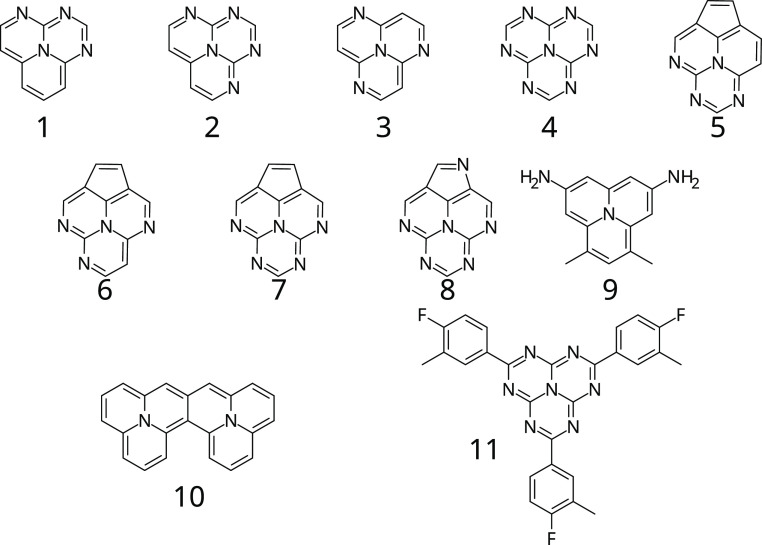
Structures of the set of molecules showing Δ*E*_ST_ ≤ 0 from the literature.

The starting point of this study is to verify that our computational procedure would be reliable enough to identify new IST candidates by computing the key quantities used in our screening for a set of 11 known IST molecules recently reported in the literature and shown in Figure 1 (all belonging to the triangulene family).^16,26,28,38^Table 1 reports the value of Δ*E*_ST_ given in literature and the computed parameters that we will use to discriminate among molecules in the data set to be screened. Unless stated otherwise, all quantum chemical calculations were carried out with the Gaussian16 software.^39^ We evaluated the HOMO–LUMO exchange integral, *K*_HL_. The results in Table 1 show that *K*_HL_ ranges between 0.1 eV ≲ *K*_HL_ ≲ 0.4 eV; this observation led us to set *K*_HL_ < 0.4 eV as upper boundary for this quantity in the screening, since we are interested in molecules with electronic properties similar to known IST dyes and, with finite computational resources, looking for low *K*_HL_ increases the chances of successfully identifying new IST molecules. The *S*_1_ and *T*_1_ states of the known IST molecules are dominated by the HOMO → LUMO transition (>95% weight), and the leading term in CIS (Configuration Interaction Singles) theory stabilizing *T*_1_ with respect to *S*_1_ is ≈ 2*K*_HL_.^40^ A small *K*_HL_ is, therefore, a prerequisite for states dominated by HOMO → LUMO transitions to display IST. The condition remains relevant if the *S*_1_ state is not dominated by a HOMO → LUMO transition because a large *K*_HL_ creates a low-energy HOMO → LUMO triplet configuration that is an upper bound for the true energy of *T*_1_. A *K*_HL_ > 0.4 eV can coexist with an IST situation only if *S*_1_ does not have a dominant HOMO → LUMO configuration and has an energy at least 0.8 eV lower than the HOMO → LUMO configuration; an uncommon situation. Clearly, we cannot entirely exclude the existence of IST molecules with higher *K*_HL_, but, so far, no molecule with such feature has been identified in the literature.

**Table 1:** HOMO–LUMO Exchange Integral *K*_HL_ and Vertical Δ*E*_ST_ (in eV) of Known IST Molecules Reported in Figure 1 Computed at Various Levels of Theory (M06-2X Refers to TDDFT Calculations) and Comparison with the Literature Data. PT2 Corrections Refer to the Method Reported in the Previous Column^a^

	*K*_HL_	Δ*E*_ST_					
molecule	CIS^b^	M06-2X^b^	CASSCF(6,6)^c^	NEVPT2^d^	CASSCF(8,8)^c^	NEVPT2^d^	Lit
1	0.230	0.237	–0.141	–0.131	–0.142	–0.114	–0.076^38^
2	0.200	0.212	0.180	–0.142	0.131	–0.185	–0.134^38^
3	0.188	0.323	–0.260	–0.024	0.096		–0.084^28^
4	0.113	0.168	0.464	–0.553	–0.573		–0.242^38^
5	0.375	0.231	–0.094	–0.362	–0.051		–0.019^26^
6	0.289	0.226	–0.172		–0.061		–0.041^26^
7	0.120	0.200	0.578		–0.123		–0.055^26^
8	0.330	0.297	0.070		–0.026		–0.017^26^
9	0.210	0.199	–0.132	–0.133	–0.167	–0.130	–0.149^28^
10	0.100	0.307	–0.190	–0.128	–0.189	–0.145	–0.089^28^
11	0.095	0.131	–0.330	0.102	–0.174	–0.426	–0.160^16^

a Missing data are due to calculations that did not converge.

b 3-21G* basis set.

c 6-31G* basis set.

d def2-TZVPP basis set.

To complete the benchmarking, we computed Δ*E*_ST_ with a set of TDDFT and multiconfigurational methods, the latter also with corrections according to second order perturbation theory. As expected,^15^ TDDFT is not able to predict Δ*E*_ST_^TDDFT^ ≤ 0, but it allows for a quick selection of molecules with electronic states of the appropriate character and not too far to possibly show singlet–triplet inversion; we see that all known IST show Δ*E*_ST_^TDDFT^ < 0.4 eV. To avoid missing good candidates in the early phases of the screening, we set a cut-off for Δ*E*_ST_^TDDFT^ of 0.5 eV for molecules to be considered further. The choice of the adopted density functional (M06-2X) is based on our previous benchmarking of *S*_1_ and *T*_1_ excitation energies on a wide set of organic molecules against experimental data and other density functionals, which allowed us to identify new singlet fission^31,37^ and TADF molecules^32,37^ based on singlet–triplet gaps. The appropriateness of this functional was also recently confirmed independently by other researchers active in the field of TADF, who carried out a comparison of a large set of functionals on ≈15 TADF dyes.^41^ The adoption of multiconfigurational methods is essential to include the effect of double excitations, whose contributions to the singlet state wavefunction could result in singlet–triplet inversion. The objective is to select the cheapest method that allows introducing electronic correlation and is able to correctly identify the IST nature of all molecules reported in Figure 1. In a screening perspective, we will not adopt any automatic scheme for the selection of the active space because this is impractical for hundreds of molecules,^42,43^ but we will carry out additional multiconfigurational calculations with active space selection, including π orbitals with an appropriate number of valence electrons, on the best candidates *a posteriori*. Adopting CASSCF(6,6)/6-31G*, about half of the molecules with known singlet–triplet inversion are incorrectly described, with significant errors on the gap, up to ≈0.6 eV. Due to the lack of active space selection, these errors might arise from its incorrect description. Results greatly improve when switching to a slightly bigger active space adopting CASSCF(8,8)/6-31G*, allowing to correctly identify almost all molecules with inverted singlet–triplet gaps. Although CASSCF(8,8)/6-31G* seems, in general, superior to the CASSCF(6,6)/6-31G*, there are some specific molecules for which the latter gives correct results, and the former does not. For this reason, in our screening procedure, we decided to compute Δ*E*_ST_ with both methods, and to further consider molecules showing Δ*E*_ST_ ≤ 0 with at least one method. Finally, we adopted the method NEVPT2^44−47^ to introduce electron correlation missed by CASSCF calculations. NEVPT2 calculations, carried out with the Orca software,^48^ identify correctly almost all IST molecules, both in case of CASSCF(6,6) and CASSCF(8,8) wavefunctions.

Before discussing the set of molecules to be screened and the results of the screening, we deem appropriate to evaluate the ability of the chosen strategy to miss potential IST candidates, with indications on how to further reduce this undesirable outcome. On the basis of 35 molecules (the 11 reported in Figure 1, 10 TADF molecules discussed by us in Table 1 of ref (32), and 14 TADF molecules discussed by others in ref (41)) where we considered a small Δ*E*_ST_ computed with TDDFT in comparison with advanced methods or experiments, we have never observed a relative stabilization of the triplet with respect to the singlet overestimated by more than 0.4 eV. This observation suggests that a larger overestimation of the singlet–triplet gap may take place for approximately 3% of the cases. There is, therefore, an extremely low chance that IST molecules will be found among molecules with TDDFT-computed Δ*E*_ST_ > 0.5 eV, and one could systematically reduce this probability by increasing this threshold if desired. The observation that all 11 known IST molecules have *K*_HL_ < 0.4 eV suggest that this in an excellent criterion for narrowing down the search. If this sample were completely random, a hypothesis that cannot be rigorously tested, we would conclude that >90% of IST molecules have *K*_HL_ < 0.4 eV but it is the physical justification presented earlier that gives the strongest support to this criterion. Collecting more data on Δ*E*_ST_*vsK*_HL_ can be helpful in determining with greater confidence the ideal cut-off. The rough CASSCF(6,6) and CASSCF(8,8) calculations have been designed not to miss any of the known IST molecules and so has the same fidelity of the *K*_HL_ criterion, with the same route for improvement, i.e., by increasing the data set.

## 3 Results and Discussion

As a next step, we built a data set of molecules to be screened with the methods applied to the known IST molecules described above. We analyzed a subset of the ZINC dataset^49^ consisting of ≈6500 molecules showing Δ*E*_ST_^TDDFT^ ≤ 0.5 eV computed at TDDFT/M06-2X/3-21G*, with geometries generated from SMILES strings^50−52^ and optimized on the ground state at the BLYP35/3-21G* level. Additionally, we analyzed a subset of the Cambridge Structural Database^53^ obtained in previous screening studies,^37^ consisting of ≈8500 molecules showing Δ*E*_ST_^TDDFT^ ≤ 0.5 eV. TDDFT calculations on this dataset were performed on the available X-ray geometries at the M06-2X/def2-SVP level to compute Δ*E*_ST_^TDDFT^. The ≈8500 promising candidates were then also optimized on the ground state at the BLYP35/3-21G* level to make the two data sets consistent with one another. We underline that the use of these (TD)DFT methods and functionals to screen for molecules based on their singlet–triplet gaps was first proposed in ref (54), and validated by us in a series of following works^31,32,37^ and, independently, by others.^41^ The union of these two subsets (ZINC and CSD) consisted of ≈15 000 molecules and was first of all filtered for rigidity,^31^ analyzing only molecules showing a number of rotatable bonds *n*_rots_ ≤ 1, as commonly done in cheminformatics screenings,^55^ and as evaluated from SMILES strings through the RDKit,^56^ without adding hydrogen atoms to the molecular graph generated from the string. This criterion was chosen due to its low computational cost, with the aim to avoid flexible molecules with potential ultrafast radiationless decay through conical intersections^57^ and where large displacements from equilibrium positions may induce instabilities in optical devices, and resulted in a total of ≈2700 molecules. Nevertheless, this condition can be relaxed in case experimental evidence of flexible IST molecules would become available.

**Figure 2:**
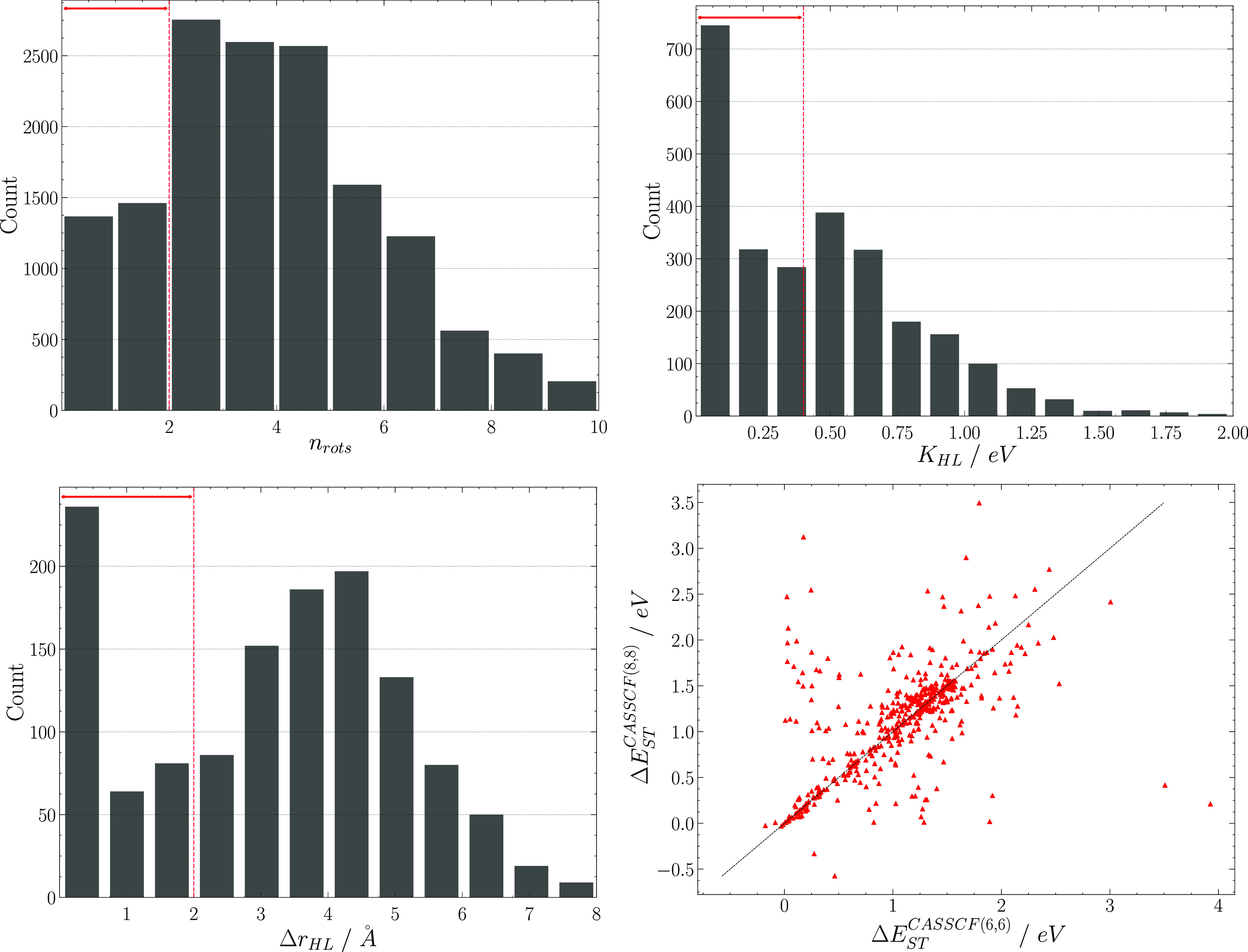
Top left: distribution of molecules with a specific number of rotatable bonds. Top right: distribution of CIS/3-21G* HOMO–LUMO exchange integrals. Bottom left: distribution of Δ*r*_HL_ metric for the HOMO → LUMO transition. Red lines identify the range within which known IST molecules fall, determining the thresholds used to select candidates. Bottom right: correlation between vertical Δ*E*_ST_ computed at CASSCF(6,6)/6-31G* and CASSCF(8,8)/6-31G* levels.

The next step in our computational funnel consisted in evaluating the HOMO–LUMO exchange integral *K*_HL_ at the CIS/3-21G* level as . For a randomly chosen subset, we evaluated the exchange interaction *K*_HL_ also with the def2-TZVP basis set; the reasonable correlation between the two sets of results (*R*^2^ = 0.87, see Supporting Information) allows for a very fast computation of *K*_HL_ with the smaller basis set, taking only 1% of the CPU time required at the higher level of theory. Of these CIS calculations, 9% failed and were not considered further, while candidates showing *K*_HL_ < 0.4 eV were further processed (≈1300 molecules, see Figure 2). The low value of *K*_HL_ in molecules displaying IST like triangulene or heptazine is due to the very limited overlap between HOMO and LUMO electronic densities, which are often described as complementary, i.e., with orbital density on different atoms. Small *K*_HL_ can also be trivially due to a strong spatial separation between frontier orbitals, leading to an excitation with charge transfer character that is not of interest for IST. We excluded from further consideration molecules with spatially separated HOMO and LUMO by computing the distance between the centroids of these orbitals, Δ*r*_HL_, with the Multiwfn software,^58^ and including molecules only if Δ*r*_HL_ < 2 Å.^59^ This constraint reduced the data set by ≈ 40%, bringing the number of molecules to consider to 760 (see Figure 2).

For these structures we computed Δ*E*_ST_^CASSCF^ at both the CASSCF(6,6)/6-31G* and CASSCF(8,8)/6-31G* levels with no intervention on the active space or orbital ordering. This crude approach with CASSCF can yield erroneous cases but serves as a necessary and useful intermediate step, being effective at identifying promising candidates, as seen in Table 1. From the CASSCF(6,6) calculations, 24% failed to converge; however, 4 molecules were found to have Δ*E*_ST_^CASSCF^ < 0 (see Table 2). Of the CASSCF(8,8) calculations, 25% failed and 6 molecules were found to have Δ*E*_ST_^CASSCF^ < 0 (see Table 2). The two sets of successful CASSCF calculations overlap for ≈90%, and the portion of unique molecules for which both CASSCF(6,6) and CASSCF(8,8) calculations fail at this stage is 18%. In Figure 2, we report the correlation between Δ*E*_ST_^CASSCF^ gaps computed at the two chosen CASSCF levels. While there are many points far from the perfect correlation line, which can be explained with the fact that we did not act on the active space in these calculations, in the region that we are focusing on (Δ*E*_ST_^CASSCF^ ≲ 0), the correspondence between the two methods is satisfactory. However, to compensate for our lack of active space adjustments, we selected all molecules where Δ*E*_ST_^CASSCF^ ≤ 0 with at least one of the two methods.

**Figure 3:**
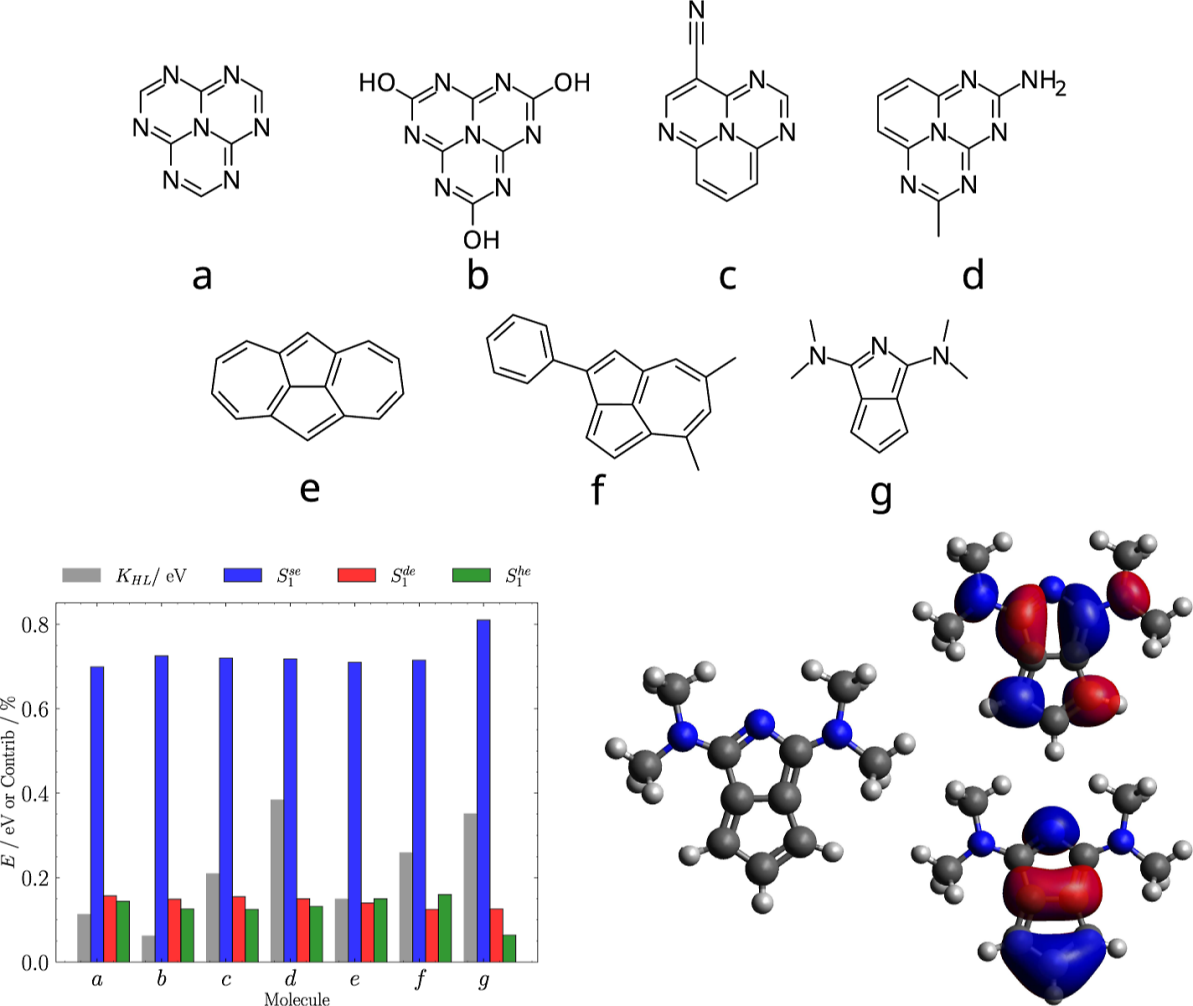
Top: structures of identified molecules showing Δ*E*_ST_ ≤ 0. Bottom left: exchange energy (*K*_HL_, in eV; gray) and fractional contribution of single excitations (*S*_1_^se^; blue), double excitations (*S*_1_^de^; red) and higher excitations (*S*_1_^he^; green) to the SA-CASSCF wavefunction of the *S*_1_ state for the identified molecules. Bottom right: frontier molecular orbitals for molecule g.

The electronic properties obtained in the screening for these molecules are summarized in Figure 3 and reported in Table 2. All 7 identified molecules show IST with at least one of the two proxy CASSCF methods, with perturbative corrections always yielding a negative Δ*E*_ST_, except for molecule *g*. Due to the very small pool of examples, which can be easily divided into families (*vide infra*), it is not straightforward to pinpoint the specific reason behind this behavior. Moreover, due to the fact that (i) ground state geometries were optimized with a small basis set, (ii) the gaps considered here are vertical gaps, (iii) the multiconfigurational methods were applied without a proper selection of active space, we proceeded with further verifications on the identified molecules before any further analysis. The adoption of more accurate calculations as the screening proceeds guarantees that false positives identified in the previous steps are safely removed. The first family (molecules *a*–*d*) are heptazine derivatives, which is unsurprising as these molecules are already known to show singlet–triplet inversion and serve as a validation of the method. Nonetheless, molecules *c* and *d* were not previously known, to the best of our knowledge, as cores to be potentially modified to achieve high oscillator strengths. The fact that molecule d has a much higher oscillator strength than the rest of the identified ones highlights how screening procedures can provide indications on the chemical modification of known cores to achieve the desired properties: symmetry breaking and an electron-donating substituent in this case provide a candidate with potentially exploitable emissive properties. Molecules (*e*–*g*) are characterized by a pentalene core. Pentalene is known to give rise to singlet–triplet inversion in its planar *D*_2h_ geometry,^60^ which is, however, antiaromatic, and thus unstable. Recently, Blaskovits identified molecule *e* (CSD identifier: COLDEM) through a screening procedure of a CSD-based data set, arguing that the planarity of the pentalene core was driven by fusion with a 7-membered ring, giving rise to an azulene dimer where the stabilization of the pentalene core is symmetry-driven and leads to singlet–triplet inversion, taking origin from the pentalene frontier orbitals.^36^ The same idea proposed by Blaskovits applies to molecule *f*, where the pentalene core is fused to a 7-membered ring. Interestingly, azulene was not identified as an IST molecule, in agreement with experiments,^61^ and as can be expected from the fact that the frontier orbitals are localized on the pentalene unit,^36^ which is not present in the azulene scaffold. Molecule *g* instead shows an isolated pentalene core where a carbon atom has been replaced with a nitrogen. Due to its novelty with respect to the other identified molecules, a more detailed analysis can reveal additional strategies to stabilize the pentalene core, allowing to design new derivatives to exploit its IST features.

**Table 2:** HOMO–LUMO Exchange Integral*K*_HL_ and Vertical Δ*E*_ST_ (in eV) of Identified IST Molecules Reported in Figure 3^a^

	*K*_HL_	Δ*E*_ST_			
molecule	CIS^b^	CASSCF(6,6)^c^	NEVPT2^d^	CASSCF(8,8)^c^	NEVPT2^d^
*a*	0.113	0.464	–0.553	–0.573	
*b*	0.062	0.274	–0.408	–0.332	–0.401
*c*	0.209	–0.176		–0.024	
*d*	0.384	–0.028	–0.237	–0.029	
*e*	0.149	0.212	–0.197	–0.429	–0.080
*f*	0.259	–0.012	–0.073	–0.013	–0.072
*g*	0.351	–0.084	0.105	0.009	0.050

a PT2 corrections refer to the method reported in the previous column. Missing data are due to calculations that did not converge.

b 3-21G* basis set.

c 6-31G* basis set.

d def2-TZVPP basis set.

In Table 3 we report results of additional calculations: we proceeded to optimize *S*_1_ and *T*_1_ geometries at TDDFT/M06-2X/def2-TZVP level with very tight convergence criteria,^62^ in analogy to what is proposed in other works in the literature,^26,28^ and evaluated their energies using multiconfigurational and multireference approaches to obtain adiabatic Δ*E*_ST_ gaps, i.e., computed from energy minima of each state. We highlight that these gaps differ from the ones reported in Table 2 due to the different geometry used to compute the energy (*S*_0_ equilibrium geometry was used in Table 2). For a quantitative comparison of equilibrium geometries of the considered states, we refer the reader to the Supporting Information. We also computed spin–orbit couplings between states involved in the accepted mechanism for triplet harvesting of energy at *T*_1_ equilibrium geometries, since that is the relevant point for the rISC mechanism. Similarly, we evaluated oscillator strengths at *S*_1_ equilibrium geometries, since that is the relevant point for the emission process. State-average-CASSCF (SA-CASSCF) and multi-state-CASPT2 (MS-CASPT2) calculations were carried out with the (Open)Molcas software,^63−65^ including 5 singlet or triplet states and adopting the ANO-L-VDZP basis set. SA-CASSCF is a flavor of CASSCF aimed at obtaining compromise wavefunctions for all states included, avoiding root-flipping problems, while MS-CASPT2 is a multireference perturbative correction to energy also able to take into account the fact that the wavefunction is not dominated by a single Slater determinant, in analogy to the previously used NEVPT2, and to introduce the effect of dynamic correlation on top of multiple SA-CASSCF wavefunctions, especially well suited for regions of the potential energy surfaces or electronic states where CASPT2 might fail.^66^ Spin–orbit couplings were computed with the (Open)Molcas software on the SA-CASSCF wavefunctions. Concerning Δ*E*_ST_, these calculations all confirm a negative gap for all identified molecules and, as expected, all these molecules show very small oscillator strengths. As shown in other studies, these cores can be modified to achieve values more appealing to the application in OLED devices.

**Table 3:** Adiabatic Δ*E*_ST_ (in eV) Computed with Selected multiconfigurational Methods Including Multireference Perturbative Corrections on TDDFT/M06-2X/def2-TZVP Geometries, along with Oscillator Strengths at the *S*_1_ Equilibrium Geometry, and Spin–Orbit Couplings between Relevant States (in cm^–1^) at the *T*_1_ Equilibrium Geometry^a^

molecule	active space	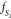	SA-CASSCF	MS-CASPT2	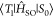	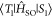
*a*	14, 13	0.0000	–0.551	–0.338	0.0042	0.0001
*b*	14, 13	0.0000	–0.439	–0.449	0.0083	0.0005
*c*	16, 15^b^	0.0007	–0.385	–0.156	0.0026	0.0006
*d*	16, 14	0.0067	–0.436	–0.022	0.0053	0.0015
*e*	16, 16^b^	0.0000	–0.325	–0.090	0.0087	0.0000
*f*	18, 18^b^	0.0003	–0.386	–0.075	0.0377	0.0031
*g*	14, 11	0.0011	–0.444	–0.117	0.1053	0.0202

a State-average and multi-state calculations included 5 states for both singlet and triplet.

b RASSCF with RAS1 = *n*_el_^act^/2 and RAS3 = *n*_orb_^act^ – RAS1, maximum 4 holes in RAS1 and maximum 4 electrons in RAS3.

We highlight how SA-CASSCF calculations provide negative gaps in all cases. This could indicate that, for the systems considered here, static correlation plays a major role for singlet–triplet inversion, although a high number of Slater determinants provides also dynamic correlation. Additionally, in other cases in the literature, the effect was mainly attributed to dynamic correlation introduced through perturbative corrections to single reference methods.^16^ We also point out that in some cases we limited the configuration interaction in the active space resorting to the RASSCF approach, to make calculations feasible on our computational infrastructure for large active spaces. In such cases, we set up the restricted active space such that excitations up to quadruples were included.

All 7 identified IST molecules retain this property at high level of theory. This can be coincidental or there may be a tendency of a preferential stabilization of the triplet. The natural continuation of this work involves the study of a few additional molecules that narrowly miss the IST criteria and establish how many should be considered further. For these reasons we have a very strong degree of confidence of the method to preselect the molecules from TDDFT and we believe that further the selection based on *K*_HL_ is reasonable and can be systematically refined. It seems unlikely that we missed molecules reaching the CASSCF(6,6)/CASSCF(8,8) level. Improvements can be introduced in selecting the best candidates from CASSCF(6,6)/CASSCF(8,8) calculations, as the current method may end up being too stringent and miss some good candidates. The list of candidates that may need reconsidering is nevertheless very short and available for further exploration.

We highlight how the newly identified core *g* shows one of the highest oscillator strengths in the set. Unsurprisingly, the  is very low as a consequence of El Sayed’s rule,^67^ thus the rISC mechanism should involve higher energy triplets to be efficient, although it shows the highest values in the set of identified molecules, making this molecule promising. The contributions of *n*th-order excitations to the *S*_1_ state of the SA-CASSCF wavefunction are also given in Figure 3, showing a clear systematic trend across the set. The double excitations (*S*_1_^de^) make up around 15% of the wavefunction, and additional higher-order excitations (*S*_1_^he^) offer a similar contribution, except for molecule g that yields only a 6% contribution from higher excitations.

The fact that molecule *g* is a completely new hit compound requires a better characterization to identify the mechanism leading to singlet–triplet inversion. We start by confirming the IST nature of this molecule with a set of other methods (see Supporting Information): we recomputed the adiabatic Δ*E*_ST_ gap from TDDFT equilibrium geometries with two different double hybrid functionals (B2PLYP-D3 and PBE0-2), and the state-of-the-art multistate perturbative method XMC-QDPT2(14,11)/6-31G*.^68,69^ In all cases, we obtain Δ*E*_ST_ < 0. Additionally, we verified the recently proposed symmetry-driven mechanism, assessing the aromaticity of molecule g by evaluating the bond-length alternation (BLA) on the main core unit at the *S*_0_ equilibrium geometry, optimized at the CASSCF(14,11)/6-31G* level, since DFT is known to poorly describe BLA, especially in comparison with multiconfigurational methods.^70^ For molecule *g*, we obtain BLA ≈0.065 Å, a value lower of that computed for several conjugated double bonds in linear chains (BLA ≈0.1 Å),^71^ which should indicate the aromatic nature of this chromophore. To provide several comparisons with known molecules optimized at the same level of theory, an aromatic molecule such as naphthalene shows BLA ≈0.05 Å, slightly lower than that of molecule g, while pentalene has BLA ≈0.15 Å. Molecule *e*, which led to the formulation of the symmetry-driven IST mechanism,^36^ shows alternating bonds of approximately the same length, with BLA ≈0.016 Å, in agreement with experiments and calculations highlighting its aromatic features.^72^ We, thus, conclude that fusion with other cores is not the only mechanism that can drive the conversion of an unstable antiaromatic core to a stable aromatic one, showing that, in case of molecule *g*, this conversion occurs by substitution of a carbon atom with a heteroatom. We highlight that the exploration of a chemical space not restricted by domain knowledge, such as the one in our starting data sets, allows the identification of unexpected candidates, which, in turn, permit refining the design rules that lead to specific properties.

## 4 Conclusions

In conclusion, we have devised a computational screening approach to extract from a very large dataset of (existing) molecules a handful of cases that display inverted energy ordering of the singlet and triplet lowest excited states. The wavefunction-based methodologies required to identify these states are computationally very expensive and generally unsuitable for virtual screening. We have, therefore, resorted to proxy properties to narrow down the search, for example, identifying molecules with small HOMO–LUMO exchange energy without charge transfer character in the lowest excited state. The final set of candidates were verified to possess inversion between singlet and triplet states with state-of-the-art methods and guarantee the correctness of the key findings (absence of false positives). Inverted singlet–triplet candidates constitute less than 0.05% of the initial set considered but contain molecules known to possess the desired characteristic, validating the screening procedure, and can be easily expanded by softening the criteria that we adopted for the search presented herein. One of the identified cores has never been reported before and can be used to expand the very limited pool of compounds with this exquisitely rare property. We have also discussed the chance that we missed candidates within the considered dataset (false negatives). They are more likely found among the ≈40 molecules showing approximate Δ*E*_ST_ ≤ 75 meV, a pool that is the ideal subject of further investigations. The new molecules identified in this work are meant to become the starting point for further investigations that are expected to cover their experimental behavior, the origin of their peculiar electronic structure, and the effect of chemical modifications on their optical properties.

## Supporting Information Available

Active space definition and occupancies, adiabatic gaps with additional single- and multi-reference methods, comparison between equilibrium geometries of *S*_0_, *S*_1_, and *T*_1_; and electronic data for molecules in the CSD and ZINC subsets, and TDDFT/M06-2X/def2-TZVP optimized geometries for identified IST molecules are available at the public GitHub repository https://github.com/dpadula85/IST_screening (PDF)
