# Supplementary material for: Identification of Unknown Inverted Singlet–Triplet Cores by High-Throughput Virtual Screening
Source: J Am Chem Soc. 2023 Aug 28;145(36):19790–9. doi: 10.1021/jacs.3c05452 (PMC10510316; doi:10.1021/jacs.3c05452)
Supplement: Supplementary file 1 — ja3c05452_si_001.pdf [file ja3c05452_si_001.pdf]

Identification of Unknown Inverted Singlet-Triplet Cores by High–Throughput Virtual Screening

Ömer H. Omar,[a] Xiaoyiu Xie,[a] Alessandro Troisi,[a*] Daniele Padula[b†]

[a] *Department of Chemistry, University of Liverpool, Liverpool, L69 3BX, U. K.*
[b] *Dipartimento di Biotecnologie, Chimica e Farmacia, Università di Siena, Via A. Moro 2, 53100 Siena, Italy*

Supporting Information

*To whom correspondence should be addressed. Email: a.troisi@liverpool.ac.uk
†To whom correspondence should be addressed. Email: daniele.padula@unisi.it

Contents

S1 Additional Figures **S5**

S2 Active Space definition **S6**

S3 Adiabatic ΔE_{ST} with selected double hybrid and multiconfigurational methods **S34**

S4 Character of TDDFT transitions at S_1 and T_1 equilibrium geometries **S35**

S5 Active space deiniftion for proxy CASSCF calculations **S39**

S6 Comparison between geometries used for vertical and adiabatic evaluations of ΔE_{ST} **S47**

List of Figures

S1 Comparison of the exchange interaction K values commputed at CIS/3-21G* versus CIS/def2-TZVP for a set of 92 molecules. S5

S2 Natural orbitals of S1 state of molecule a at S1 geometry. Numbers in figure are occupation numbers. (Package luscus[?] is used for plot) S6

S3 Natural orbitals of T1 state of molecule a at S1 geometry. Numbers in figure are occupation numbers. S7

S4 Natural orbitals of S1 state of molecule a at T1 geometry. Numbers in figure are occupation numbers. S8

S5 Natural orbitals of T1 state of molecule a at T1 geometry. Numbers in figure are occupation numbers. S9

S6 Natural orbitals of S1 state of molecule b at S1 geometry. Numbers in figure are occupation numbers. S10

S7 Natural orbitals of T1 state of molecule b at S1 geometry. Numbers in figure are occupation numbers. S11

S8 Natural orbitals of S1 state of molecule b at T1 geometry. Numbers in figure are occupation numbers. S12

S9 Natural orbitals of T1 state of molecule b at T1 geometry. Numbers in figure are occupation numbers. S13

S10 Natural orbitals of S1 state of molecule c at S1 geometry. Numbers in figure are occupation numbers. S14

S11 Natural orbitals of T1 state of molecule c at S1 geometry. Numbers in figure are occupation numbers. S15
S12 Natural orbitals of S1 state of molecule c at T1 geometry. Numbers in figure are occupation numbers. S16
S13 Natural orbitals of T1 state of molecule c at T1 geometry. Numbers in figure are occupation numbers. S17
S14 Natural orbitals of S1 state of molecule d at S1 geometry. Numbers in figure are occupation numbers. S18
S15 Natural orbitals of T1 state of molecule d at S1 geometry. Numbers in figure are occupation numbers. S19
S16 Natural orbitals of S1 state of molecule d at T1 geometry. Numbers in figure are occupation numbers. S20
S17 Natural orbitals of T1 state of molecule d at T1 geometry. Numbers in figure are occupation numbers. S21
S18 Natural orbitals of S1 state of molecule e at S1 geometry. Numbers in figure are occupation numbers. S22
S19 Natural orbitals of T1 state of molecule e at S1 geometry. Numbers in figure are occupation numbers. S23
S20 Natural orbitals of S1 state of molecule e at T1 geometry. Numbers in figure are occupation numbers. S24
S21 Natural orbitals of T1 state of molecule e at T1 geometry. Numbers in figure are occupation numbers. S25
S22 Natural orbitals of S1 state of molecule f at S1 geometry. Numbers in figure are occupation numbers. S26
S23 Natural orbitals of T1 state of molecule f at S1 geometry. Numbers in figure are occupation numbers. S27
S24 Natural orbitals of S1 state of molecule f at T1 geometry. Numbers in figure are occupation numbers. S28
S25 Natural orbitals of T1 state of molecule f at T1 geometry. Numbers in figure are occupation numbers. S29
S26 Natural orbitals of S1 state of molecule g at S1 geometry. Numbers in figure are occupation numbers. S30
S27 Natural orbitals of T1 state of molecule g at S1 geometry. Numbers in figure are occupation numbers. S31
S28 Natural orbitals of S1 state of molecule g at T1 geometry. Numbers in figure are occupation numbers. S32

S29 Natural orbitals of T1 state of molecule g at T1 geometry. Numbers in figure are occupation numbers. . . . S33
S30 HF orbitals used in CASSCF for molecule 1 . . . S39
S31 HF orbitals used in CASSCF for molecule 2 . . . S40
S32 HF orbitals used in CASSCF for molecule 3 . . . S40
S33 HF orbitals used in CASSCF for molecule 4 . . . S41
S34 HF orbitals used in CASSCF for molecule 5 . . . S42
S35 HF orbitals used in CASSCF for molecule 6 . . . S43
S36 HF orbitals used in CASSCF for molecule 7 . . . S44
S37 HF orbitals used in CASSCF for molecule 8 . . . S45
S38 HF orbitals used in CASSCF for molecule 9 . . . S46
S39 HF orbitals used in CASSCF for molecule 10 . . . S46
S40 HF orbitals used in CASSCF for molecule 11 . . . S47

S1 Additional Figures

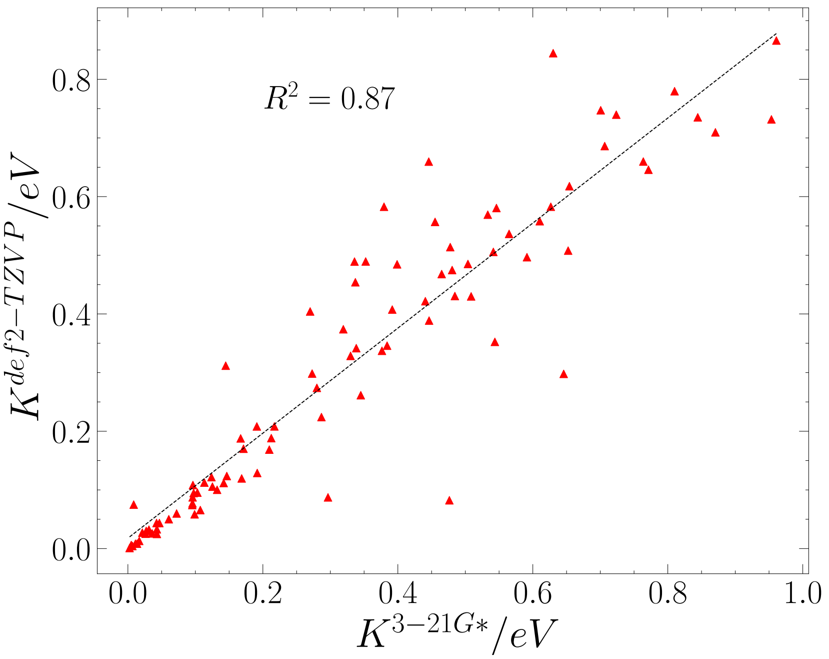

Fig. S1: Comparison of the exchange interaction K values commputed at CIS/3-21G* versus CIS/def2-TZVP for a set of 92 molecules.

S2 Active Space definition

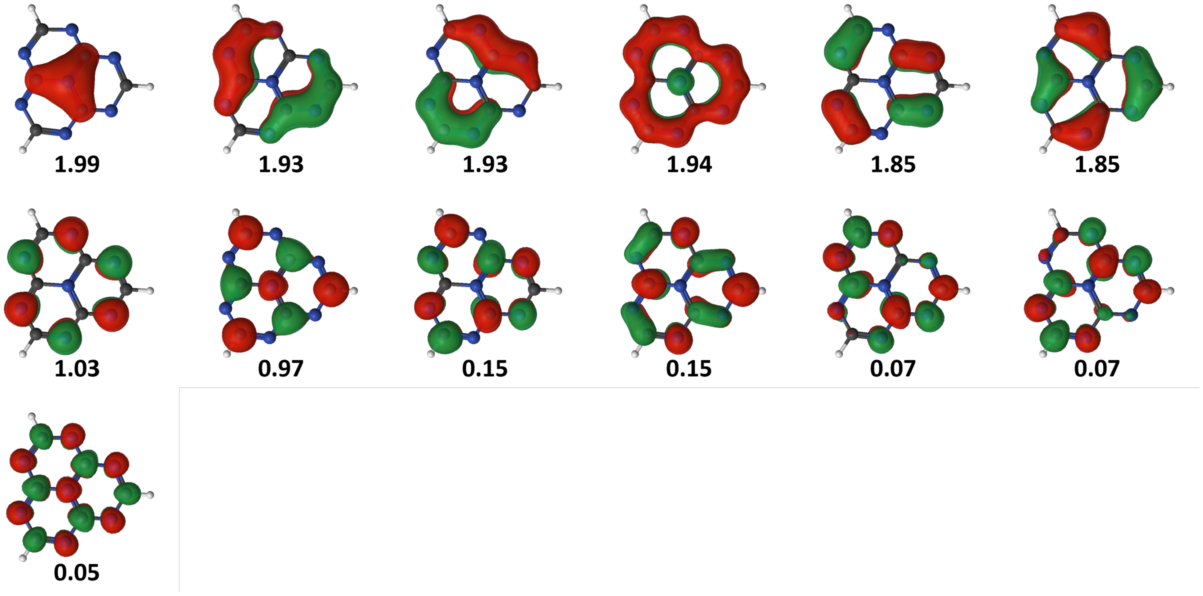

Fig. S2: Natural orbitals of S1 state of molecule a at S1 geometry. Numbers in figure are occupation numbers. (Package luscus[?] is used for plot)

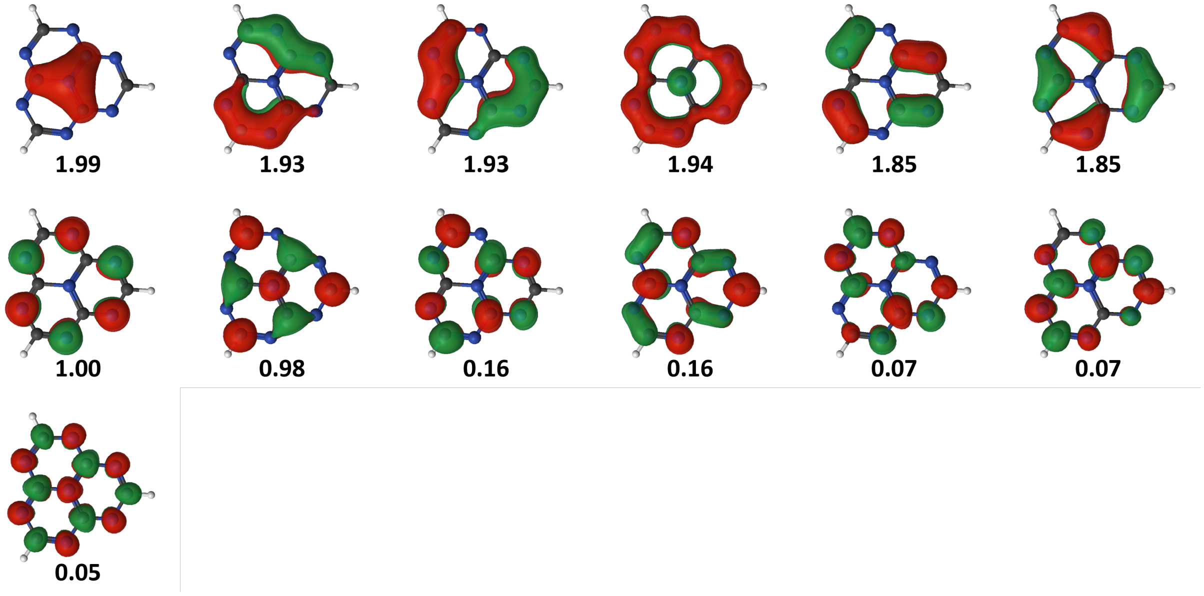

Fig. S3: Natural orbitals of T1 state of molecule a at S1 geometry. Numbers in figure are occupation numbers.

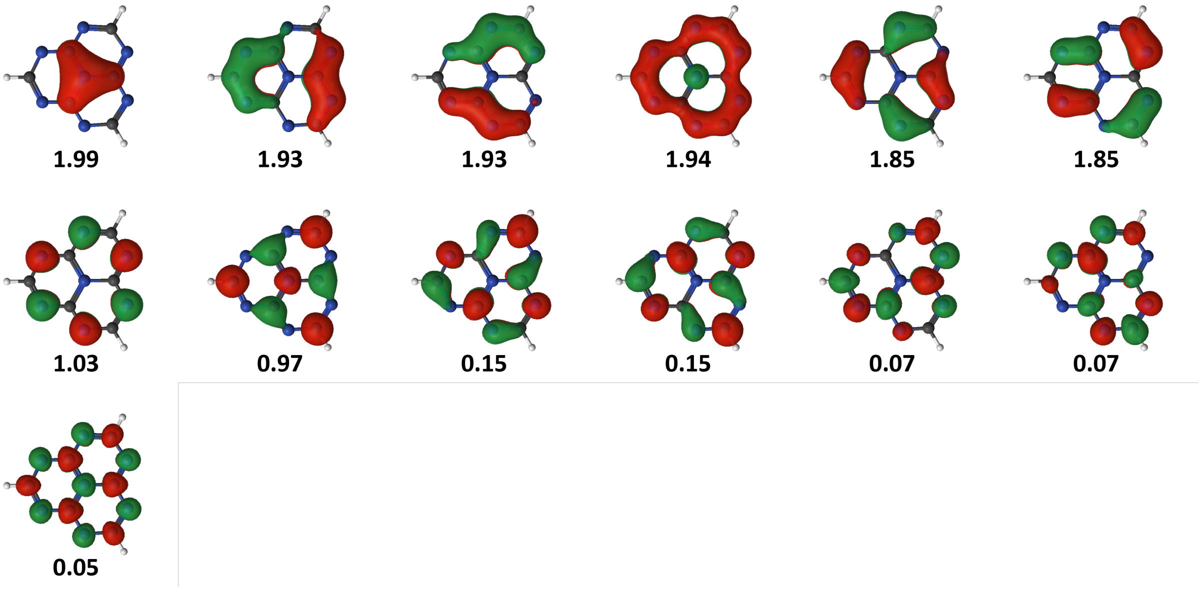

Fig. S4: Natural orbitals of S1 state of molecule a at T1 geometry. Numbers in figure are occupation numbers.

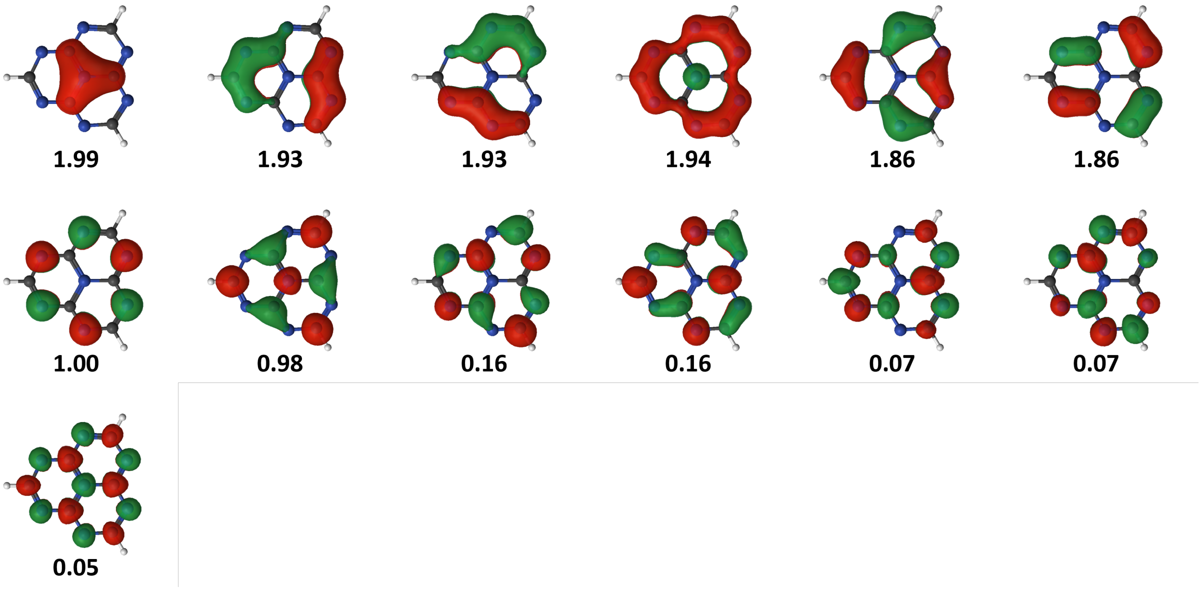

Fig. S5: Natural orbitals of T1 state of molecule a at T1 geometry. Numbers in figure are occupation numbers.

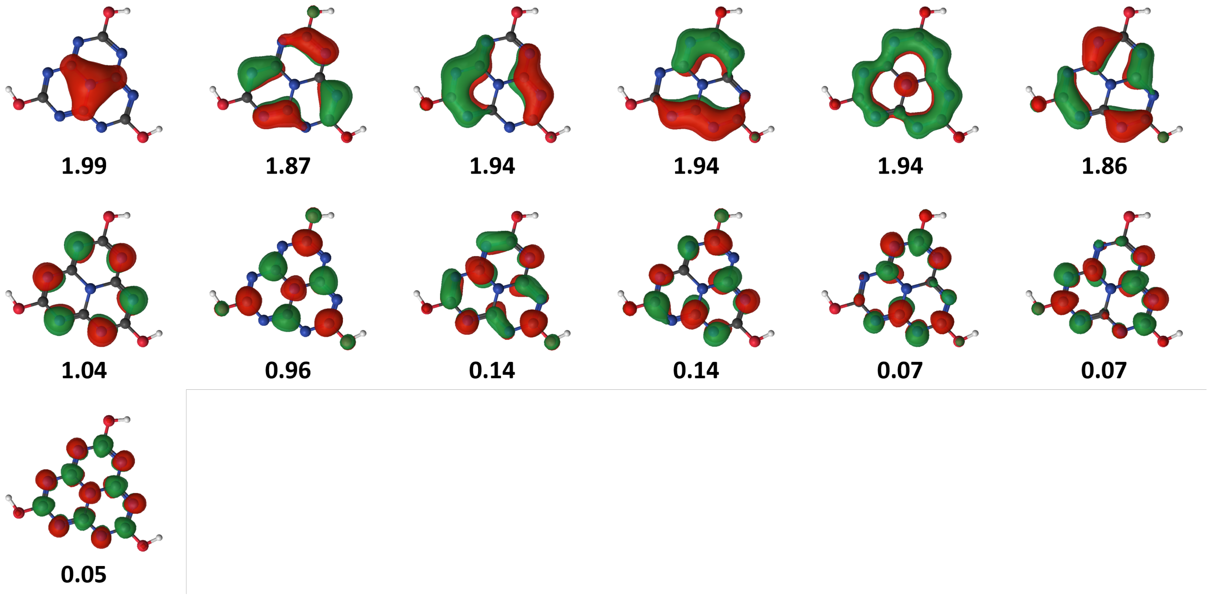

Fig. S6: Natural orbitals of S1 state of molecule b at S1 geometry. Numbers in figure are occupation numbers.

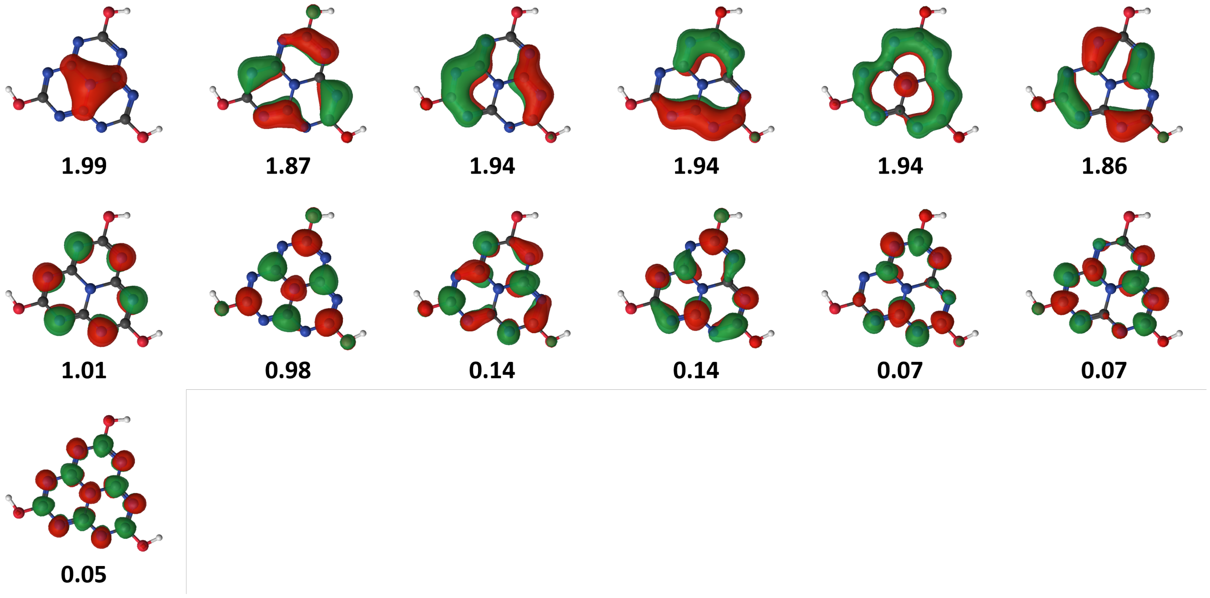

Fig. S7: Natural orbitals of T1 state of molecule b at S1 geometry. Numbers in figure are occupation numbers.

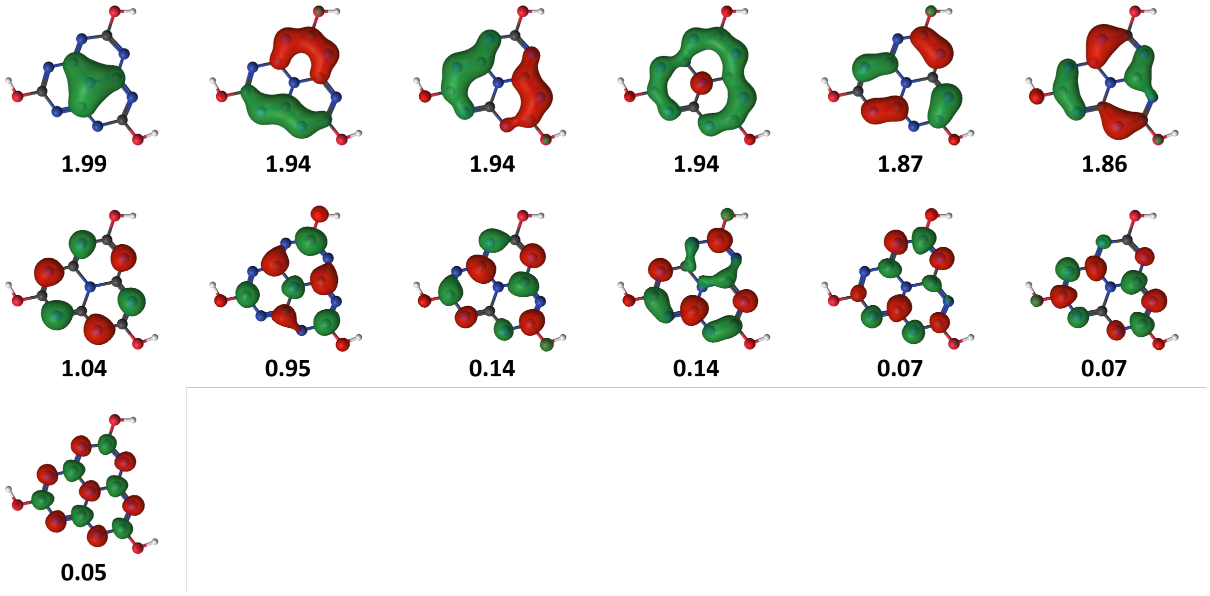

Fig. S8: Natural orbitals of S1 state of molecule b at T1 geometry. Numbers in figure are occupation numbers.

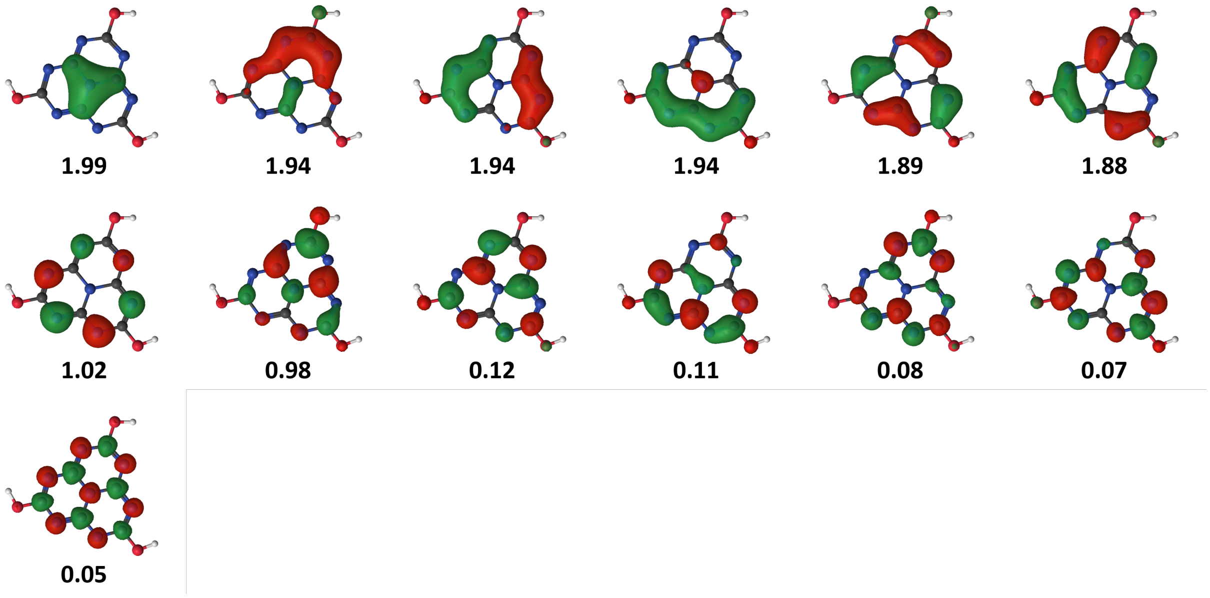

Fig. S9: Natural orbitals of T1 state of molecule b at T1 geometry. Numbers in figure are occupation numbers.

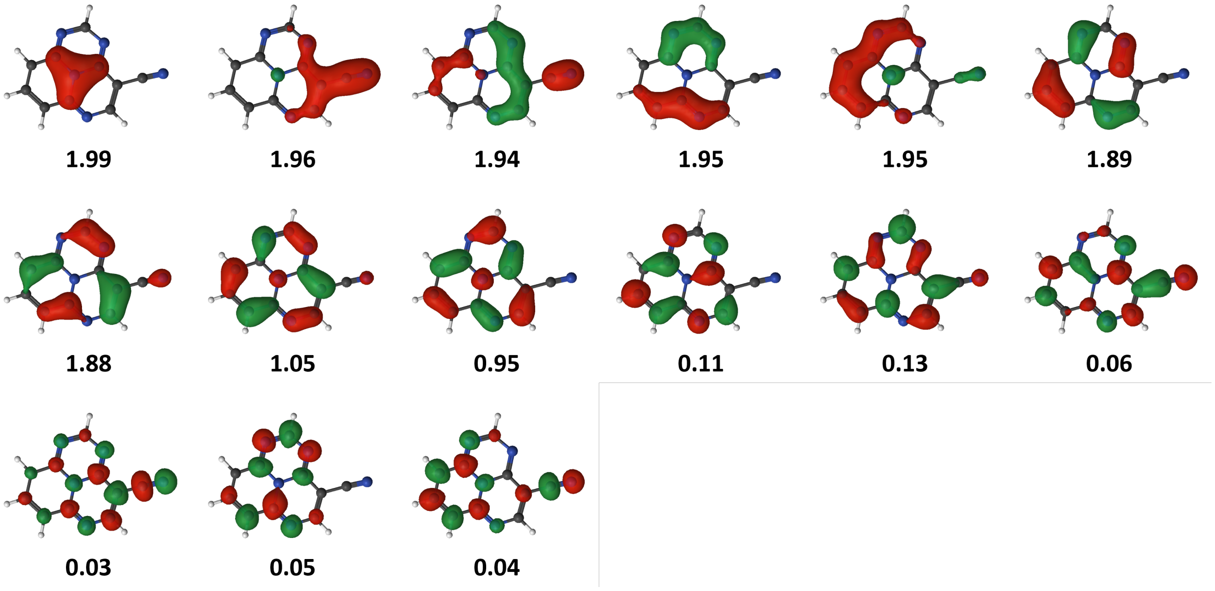

Fig. S10: Natural orbitals of S1 state of molecule c at S1 geometry. Numbers in figure are occupation numbers.

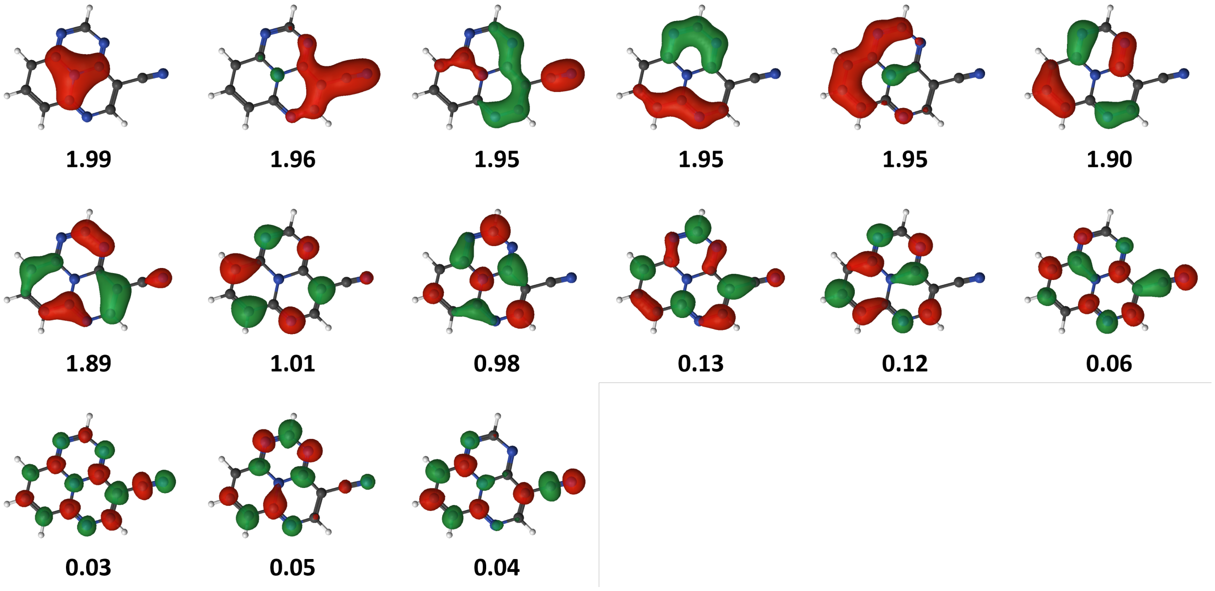

Fig. S11: Natural orbitals of T1 state of molecule c at S1 geometry. Numbers in figure are occupation numbers.

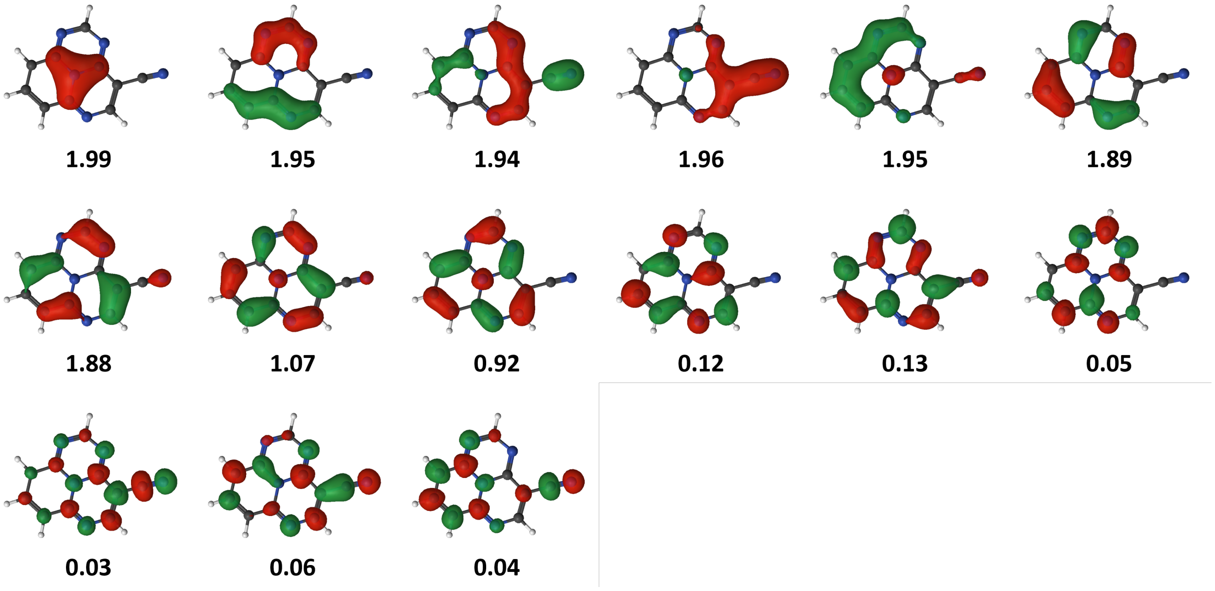

Fig. S12: Natural orbitals of S1 state of molecule c at T1 geometry. Numbers in figure are occupation numbers.

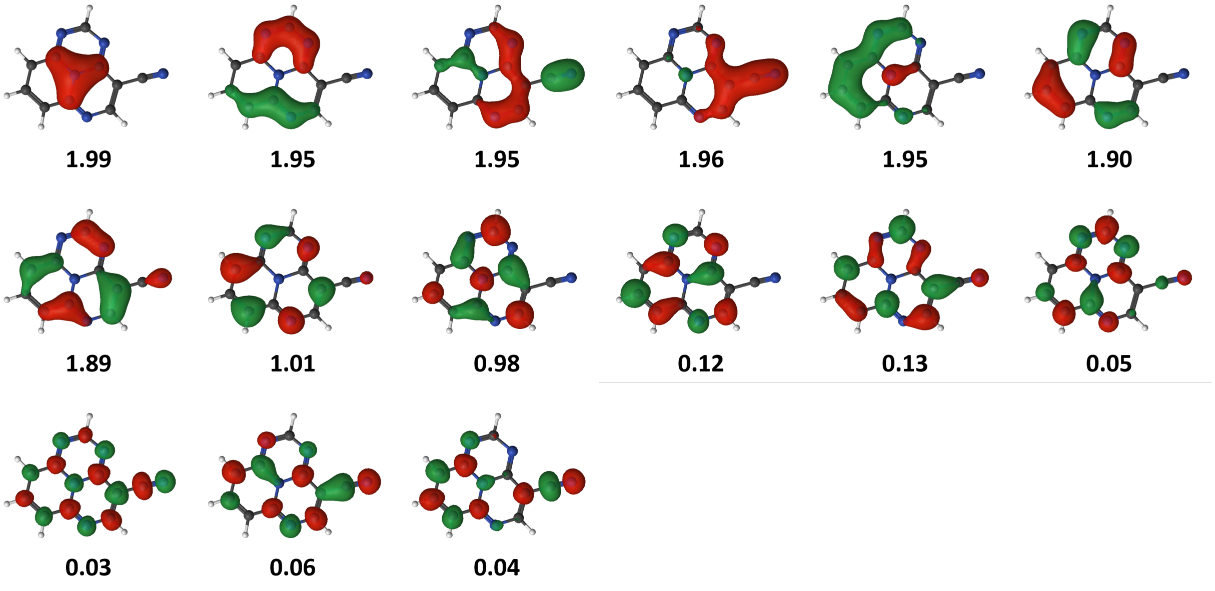

Fig. S13: Natural orbitals of T1 state of molecule c at T1 geometry. Numbers in figure are occupation numbers.

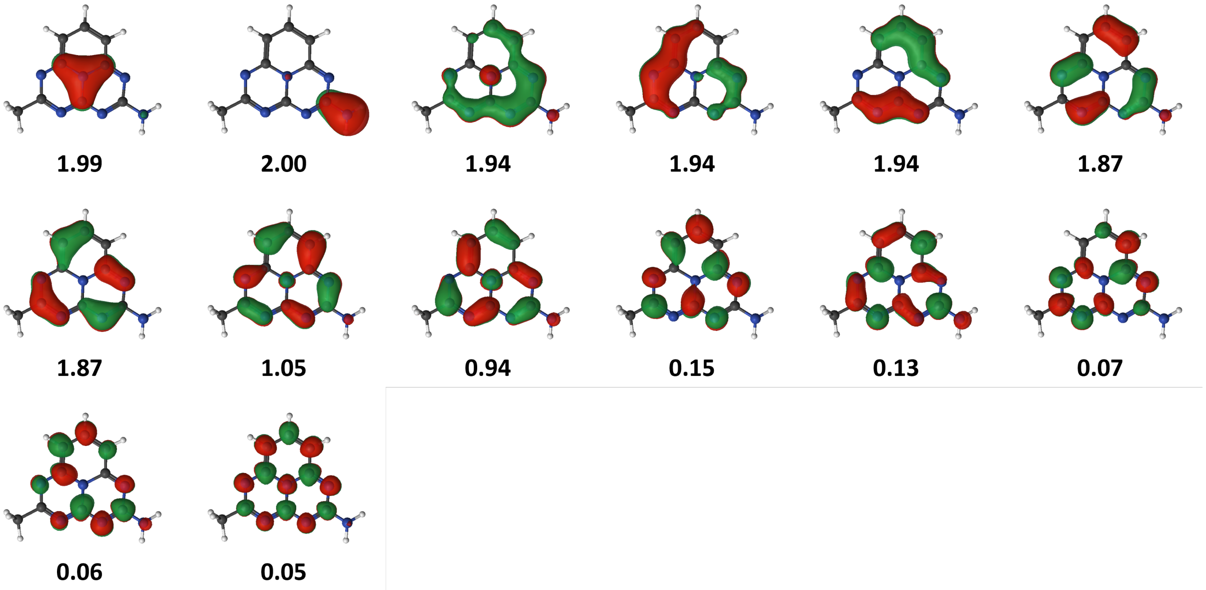

Fig. S14: Natural orbitals of S1 state of molecule d at S1 geometry. Numbers in figure are occupation numbers.

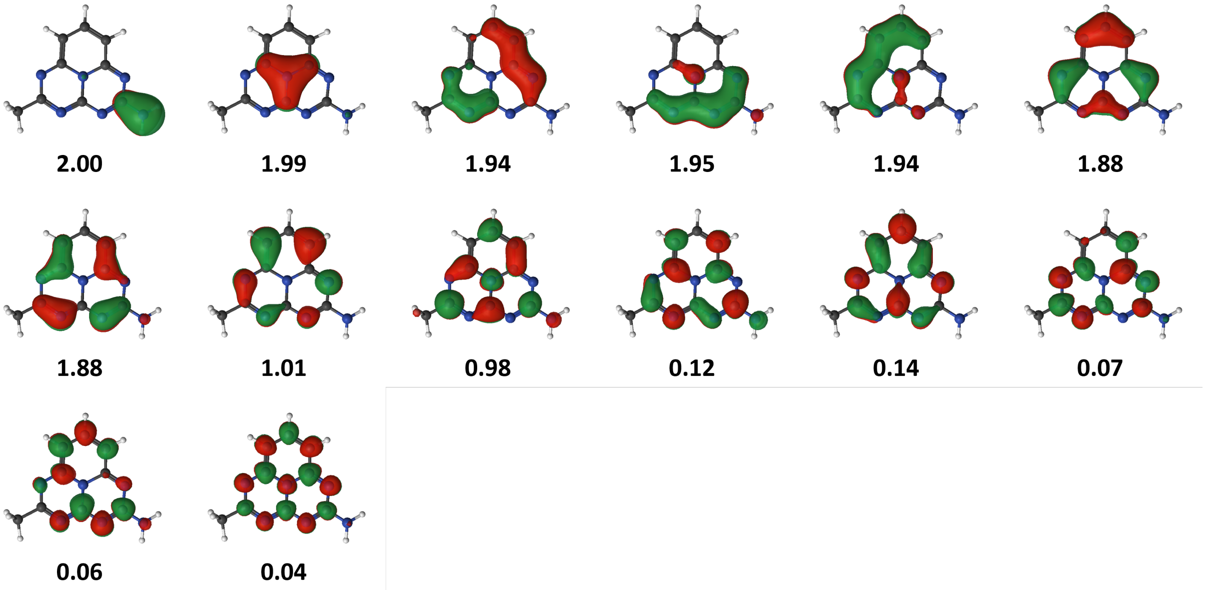

Fig. S15: Natural orbitals of T1 state of molecule d at S1 geometry. Numbers in figure are occupation numbers.

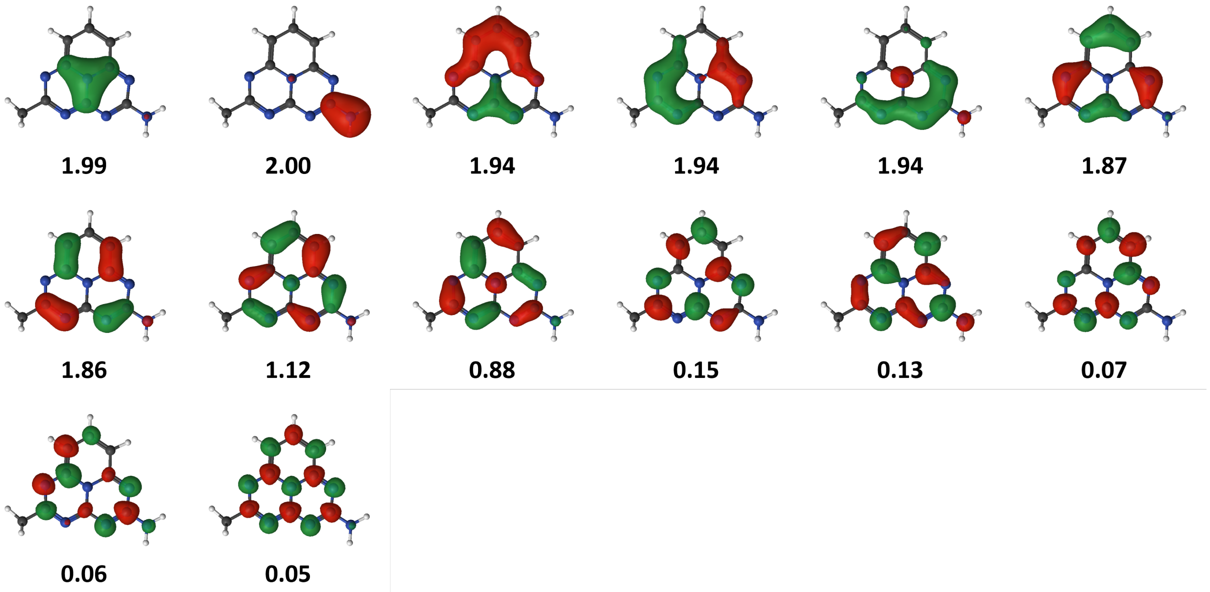

Fig. S16: Natural orbitals of S1 state of molecule d at T1 geometry. Numbers in figure are occupation numbers.

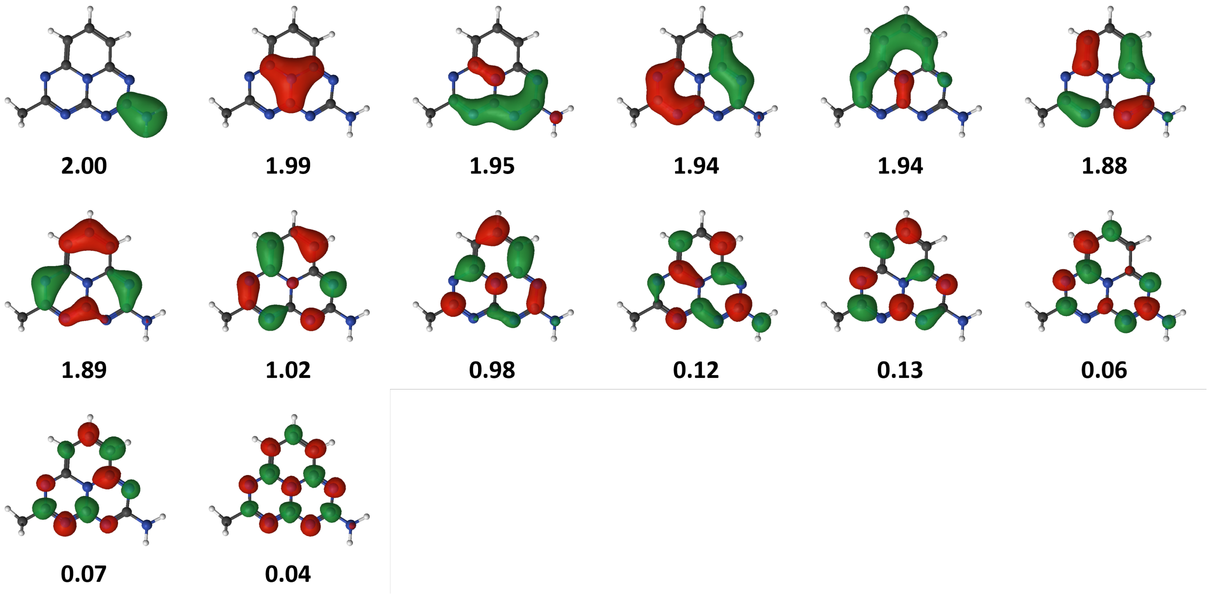

Fig. S17: Natural orbitals of T1 state of molecule d at T1 geometry. Numbers in figure are occupation numbers.

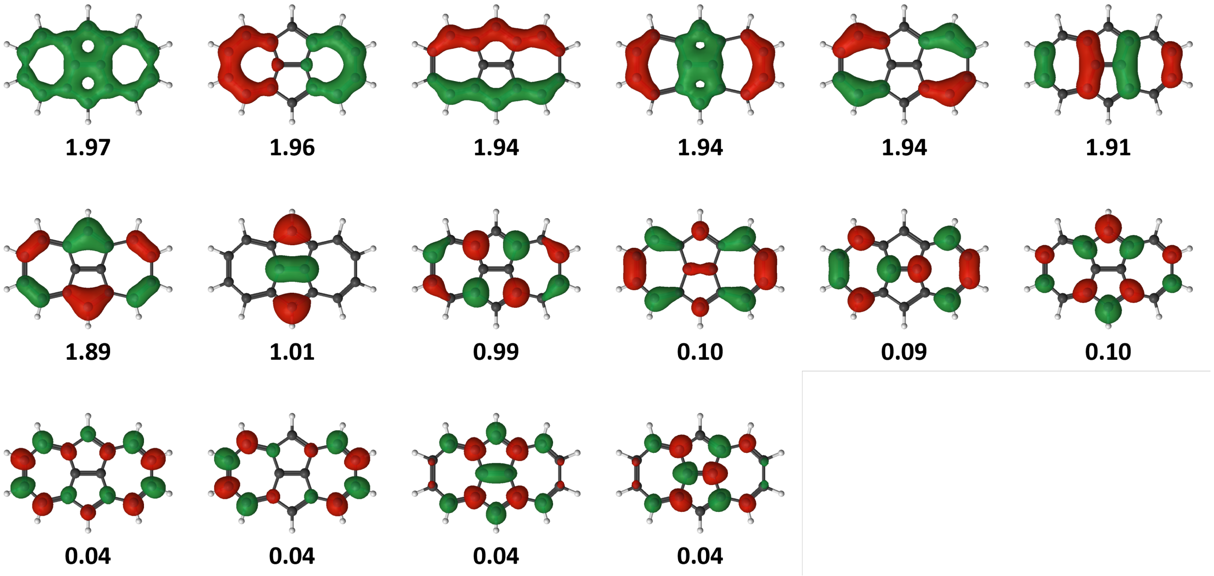

Fig. S18: Natural orbitals of S1 state of molecule e at S1 geometry. Numbers in figure are occupation numbers.

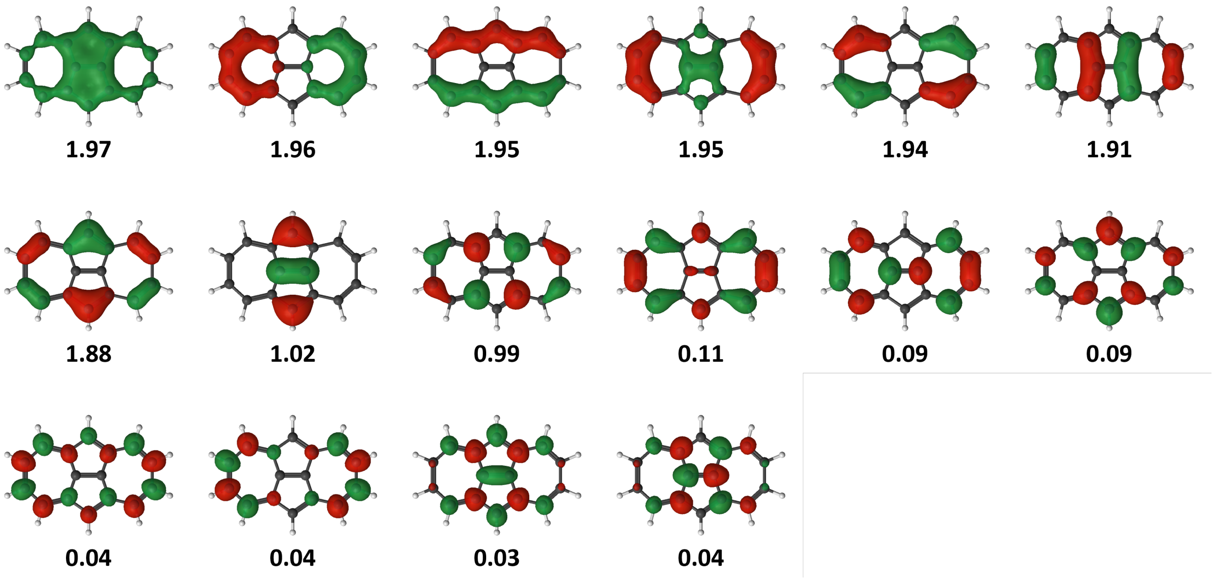

Fig. S19: Natural orbitals of T1 state of molecule e at S1 geometry. Numbers in figure are occupation numbers.

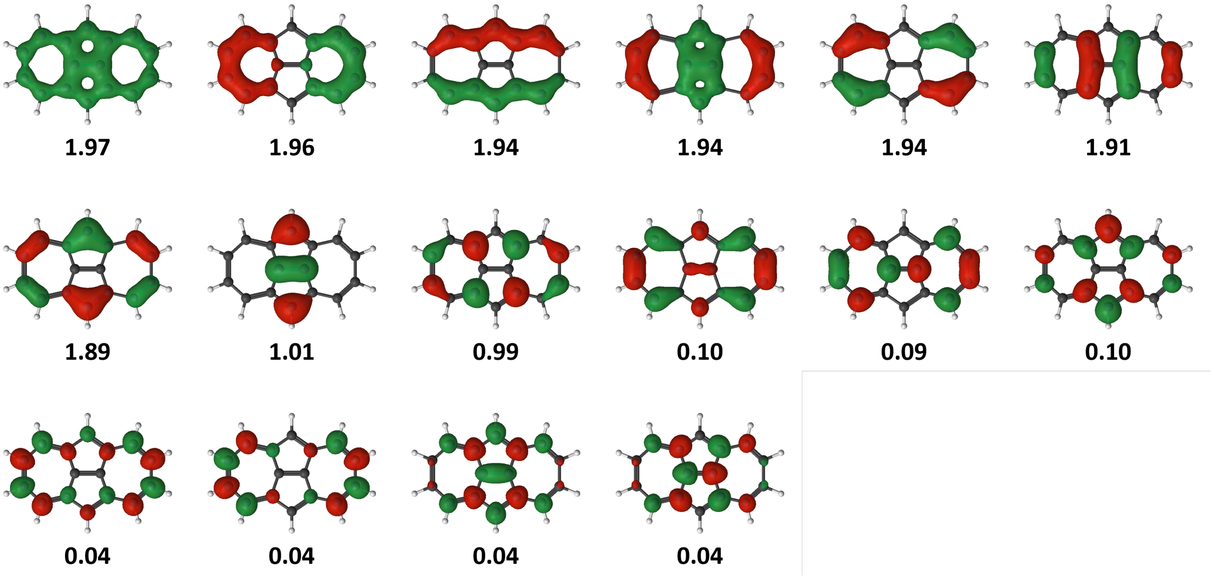

Fig. S20: Natural orbitals of S1 state of molecule e at T1 geometry. Numbers in figure are occupation numbers.

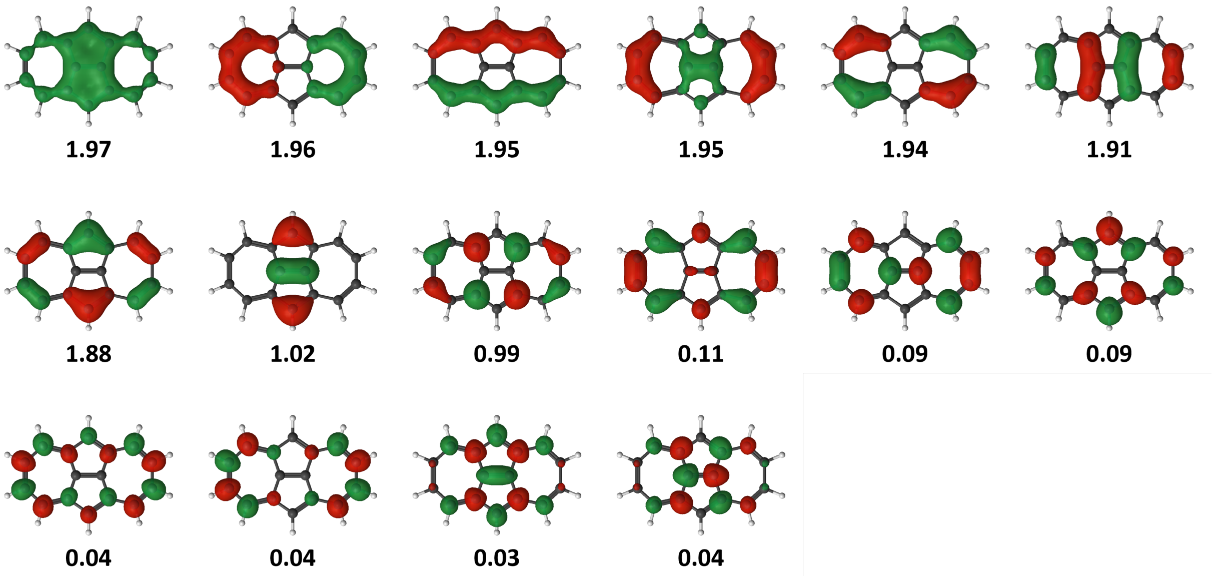

Fig. S21: Natural orbitals of T1 state of molecule e at T1 geometry. Numbers in figure are occupation numbers.

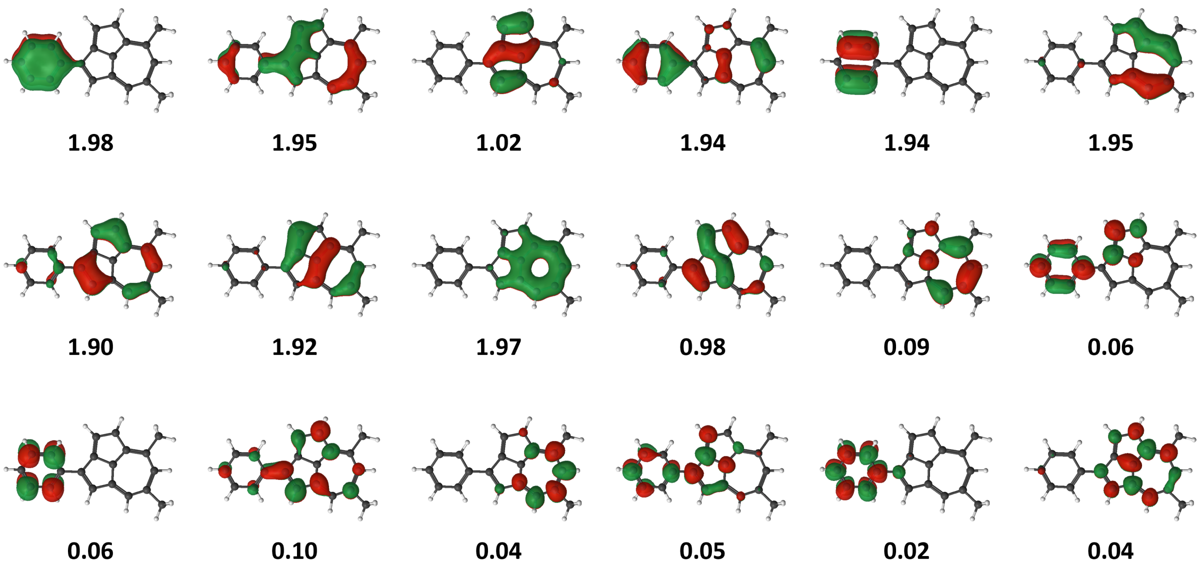

Fig. S22: Natural orbitals of S1 state of molecule f at S1 geometry. Numbers in figure are occupation numbers.

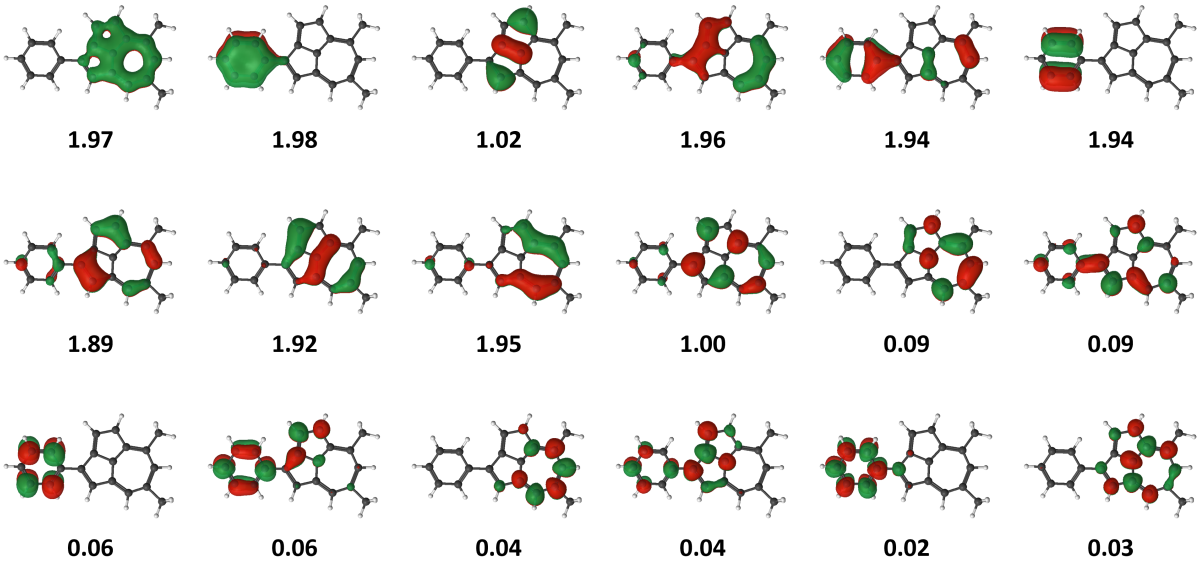

Fig. S23: Natural orbitals of T1 state of molecule f at S1 geometry. Numbers in figure are occupation numbers.

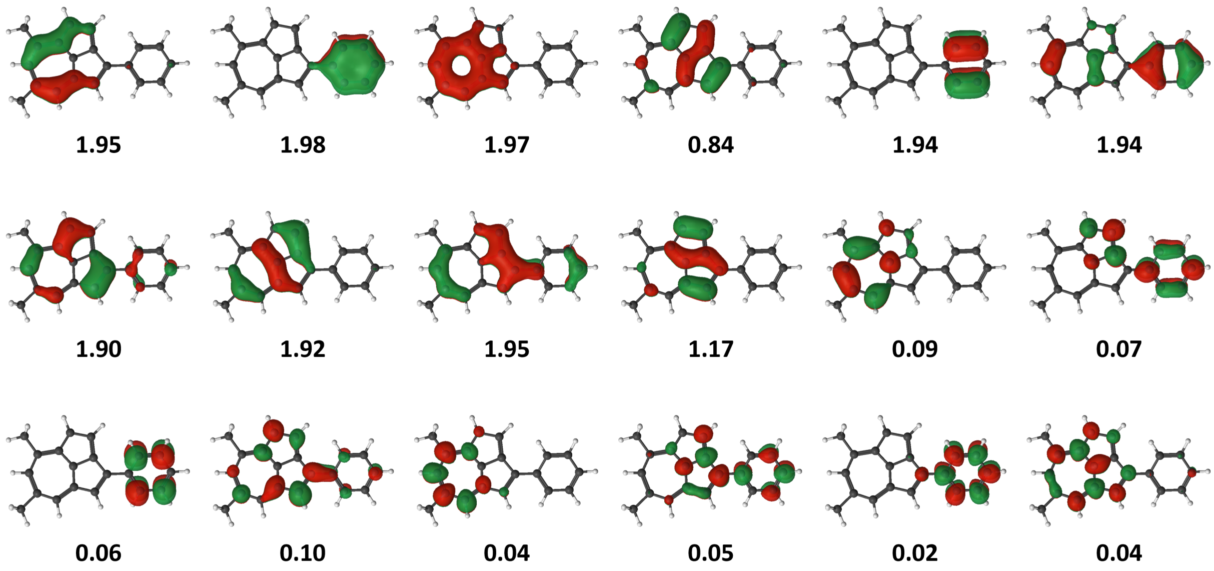

Fig. S24: Natural orbitals of S1 state of molecule f at T1 geometry. Numbers in figure are occupation numbers.

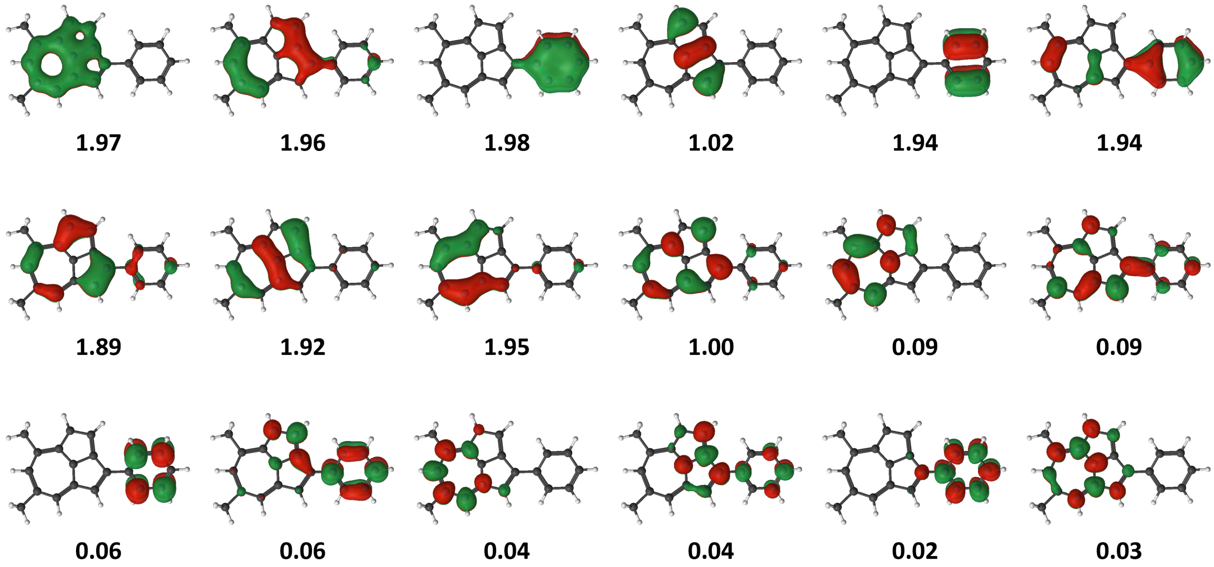

Fig. S25: Natural orbitals of T1 state of molecule f at T1 geometry. Numbers in figure are occupation numbers.

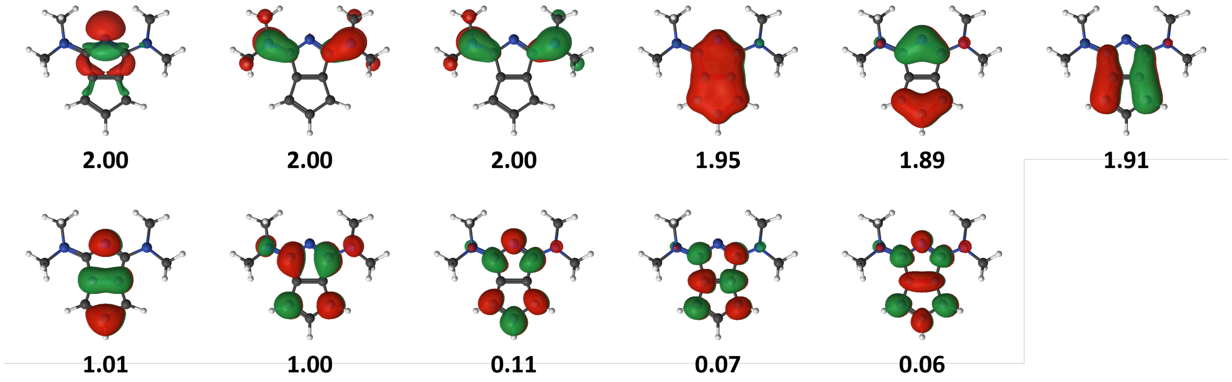

Fig. S26: Natural orbitals of S1 state of molecule g at S1 geometry. Numbers in figure are occupation numbers.

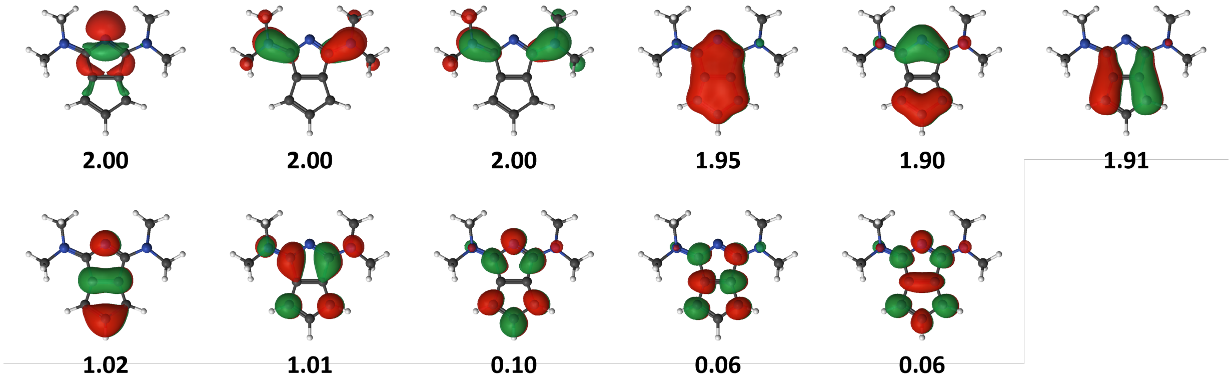

Fig. S27: Natural orbitals of T1 state of molecule g at S1 geometry. Numbers in figure are occupation numbers.

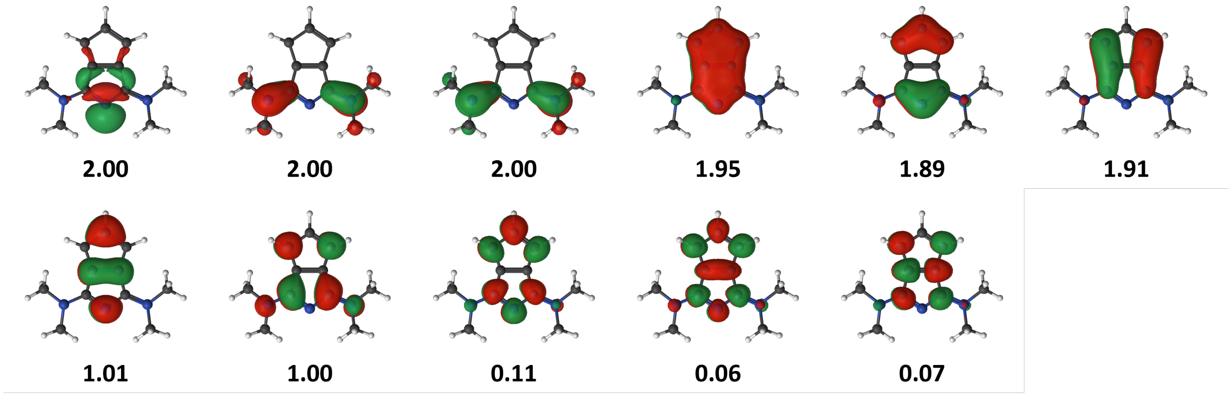

Fig. S28: Natural orbitals of S1 state of molecule g at T1 geometry. Numbers in figure are occupation numbers.

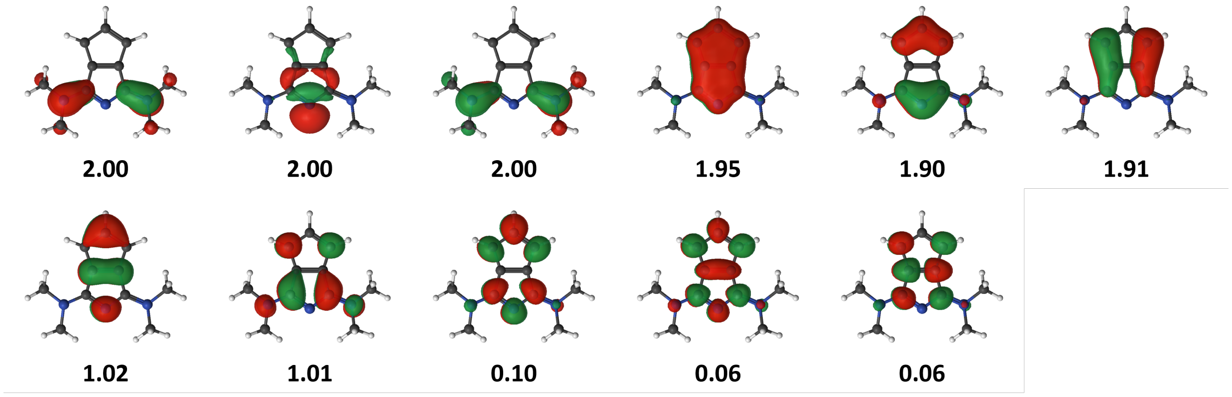

Fig. S29: Natural orbitals of T1 state of molecule g at T1 geometry. Numbers in figure are occupation numbers.

S3 Adiabatic ΔE_{ST} with selected double hybrid and multiconfigurational methods

Table S1: Adiabatic ΔE_{ST} (in eV) computed with selected methods on TDDFT/M06-2X/def2-TZVP geometries. State–averaged CASSCF performed with 4 orbitals and 4 electrons. XMC–QDPT2 calculations performed with the same active space reported in Table 3 in the main text, and 6-31G* basis set. State–averaged and multi–state calculations included 5 states for both singlet and triplet. Double-hybrid DFT performed with linear-response full TD-DFT using the RIJCOSX approximation and def2-TZVP basis set with def2/J and def2-TZVP/C auxiliary basis sets.

Molecule	SA–CASSCF	MS–CASPT2	XMC–QDPT2	B2PLYP–D3	PBE0–2
a	−0.079	−0.477		0.001	0.003
e	0.118	−0.327		0.003	0.004
g	0.375	−0.367	−0.199	−0.116	−0.124

S4 Character of TDDFT transitions at S_1 and T_1 equilibrium geometries

Molecule	Orbitals from singlet fchk	Orbitals from triplet fchk	S1 composition	T1 composition
a	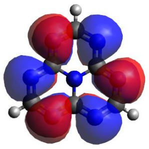 44 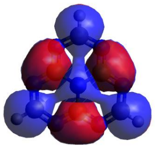 45	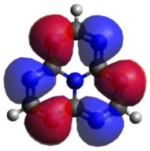 44 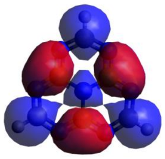 45	44 -> 45 0.69994	44 -> 45 0.69807
b	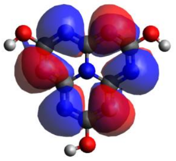 56 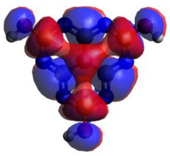 57	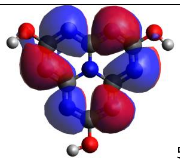 56 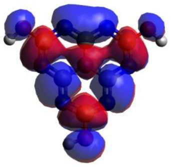 57	56 -> 57 0.69425	56 -> 57 0.67927

c	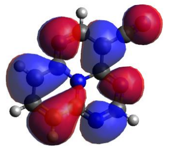 50<br>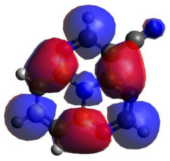 51	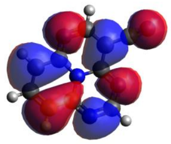 50<br>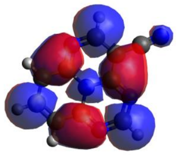 51	50 -> 51 0.70178	50 -> 51 0.70145
d	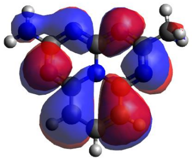 52<br>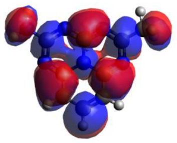 53	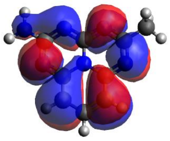 52<br>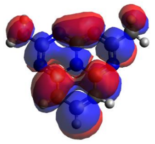 53<br>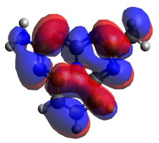 54	52 -> 53 0.69466	52 -> 53 0.69520<br>52 -> 54 0.10424

e	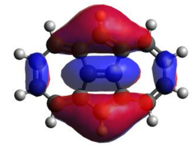 53<br>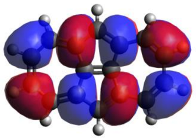 54	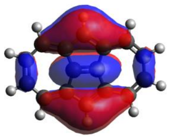 53<br>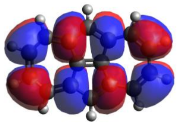 54	53 -> 54 -0.70521	53 -> 54 0.70555
f	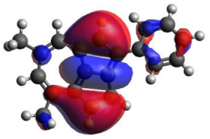 68<br>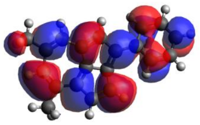 69	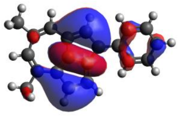 68<br>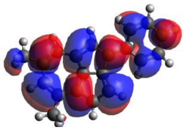 69	68 -> 69 0.70307	68 -> 69 0.70347

g	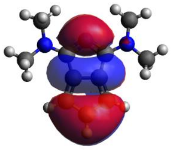 51<br>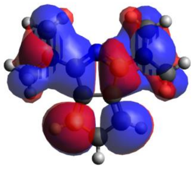 52	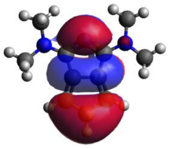 51<br>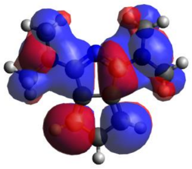 52	51 -> 52 0.70045	51 -> 52 0.70477

S5 Active space deiniftion for proxy CASSCF calculations

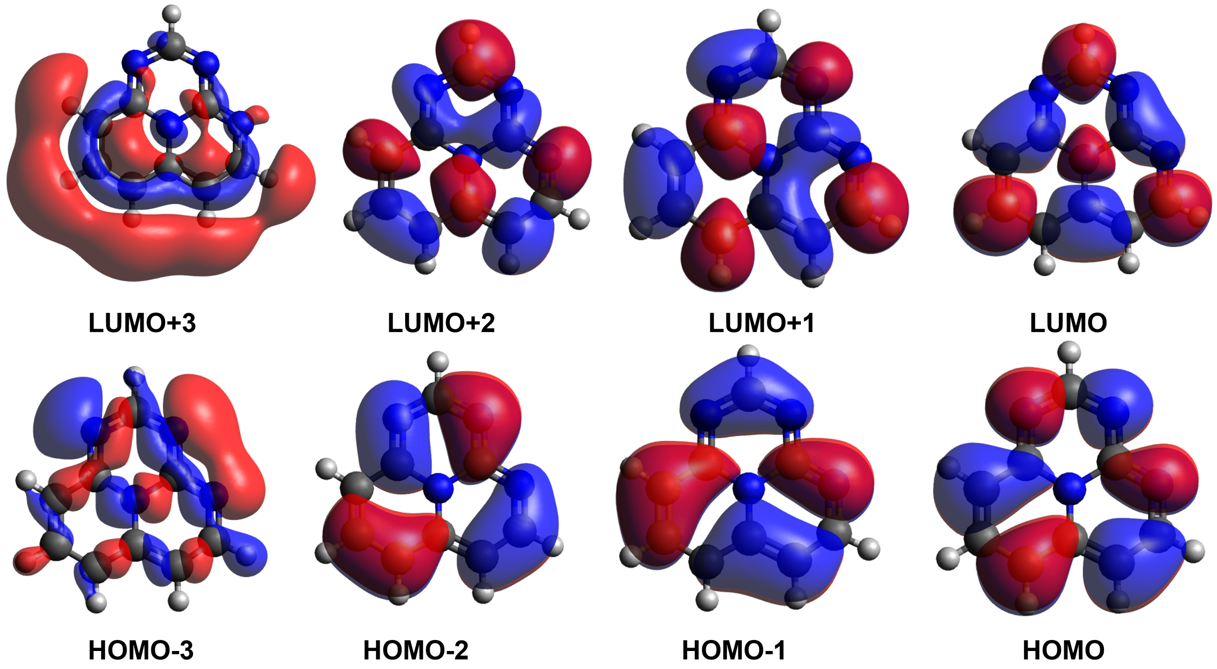

Fig. S30: HF orbitals used in CASSCF of known molecule 1. Isosurface value = 0.015.

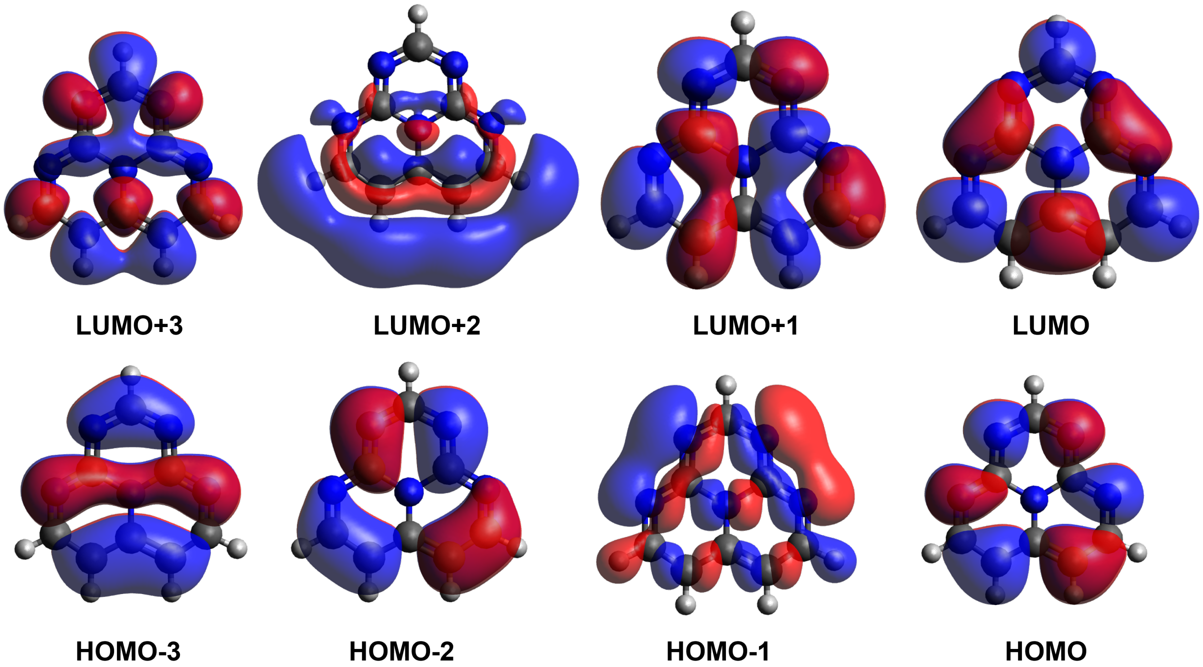

Fig. S31: HF orbitals used in CASSCF of known molecule 2. Isosurface value = 0.015.

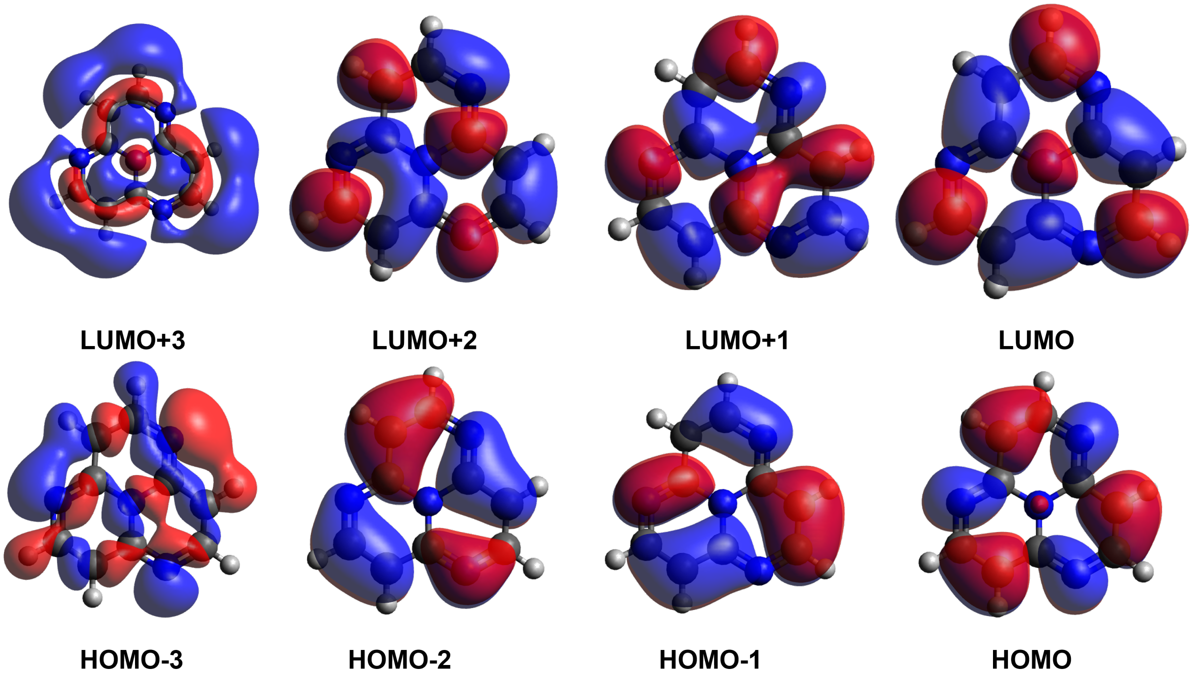

Fig. S32: HF orbitals used in CASSCF of known molecule 3. Isosurface value = 0.015.

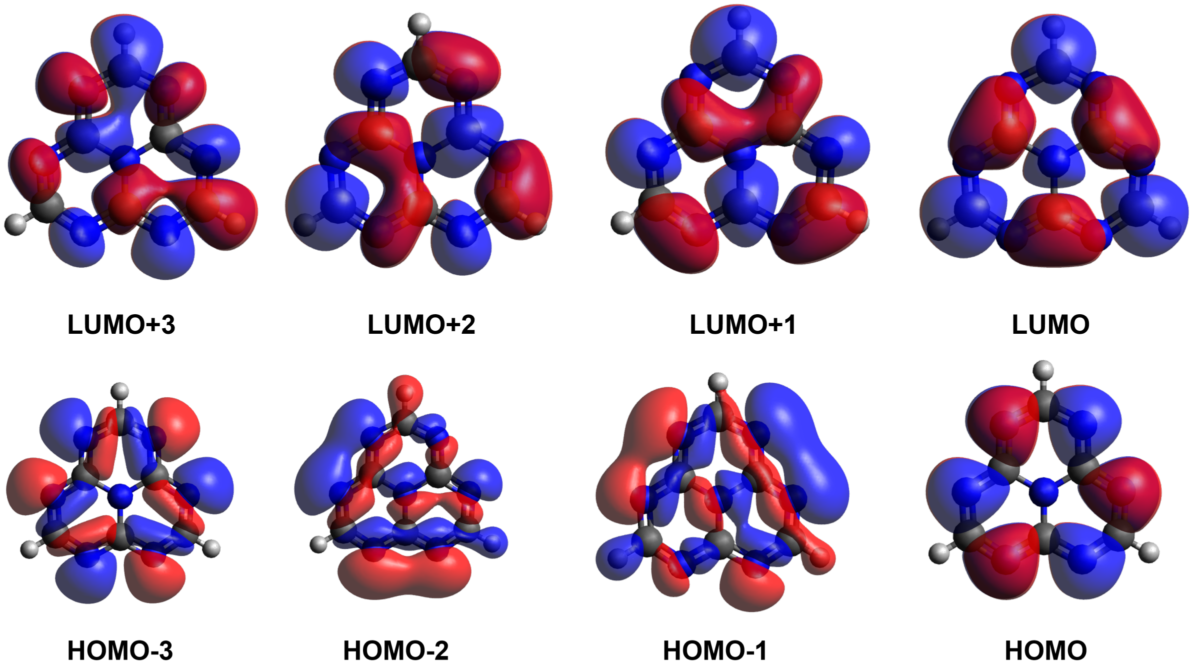

Fig. S33: HF orbitals used in CASSCF of known molecule 4. Isosurface value = 0.015.

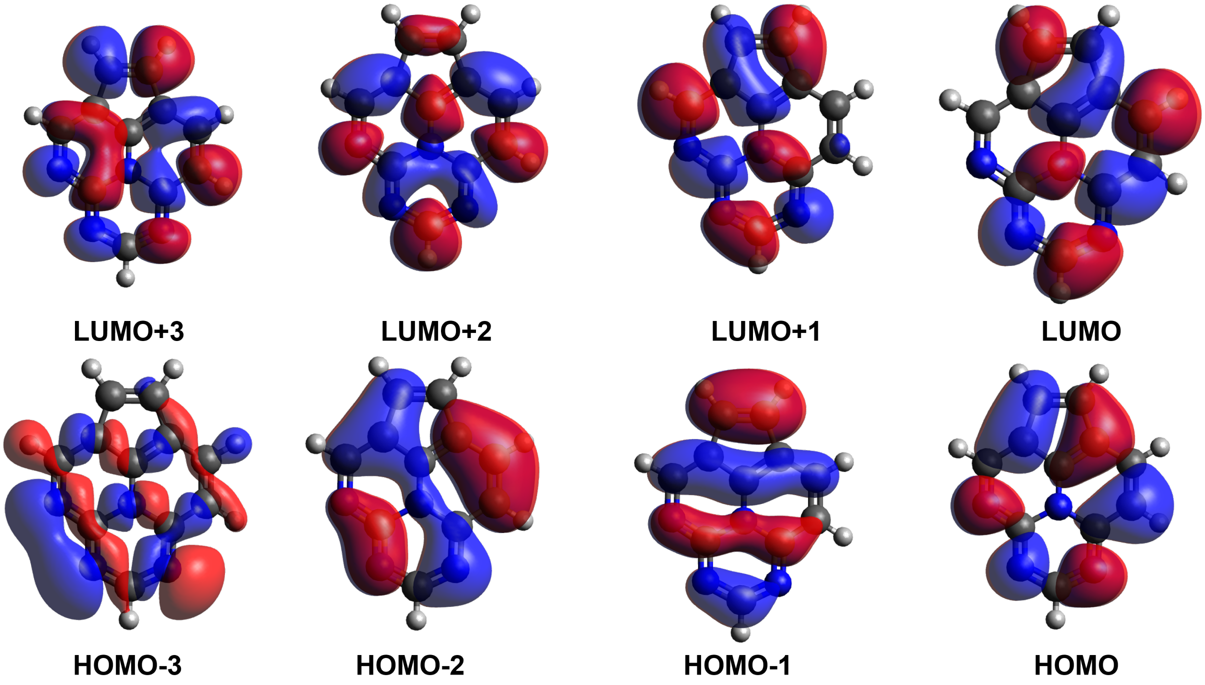

Fig. S34: HF orbitals used in CASSCF of known molecule 5. Isosurface value = 0.015.

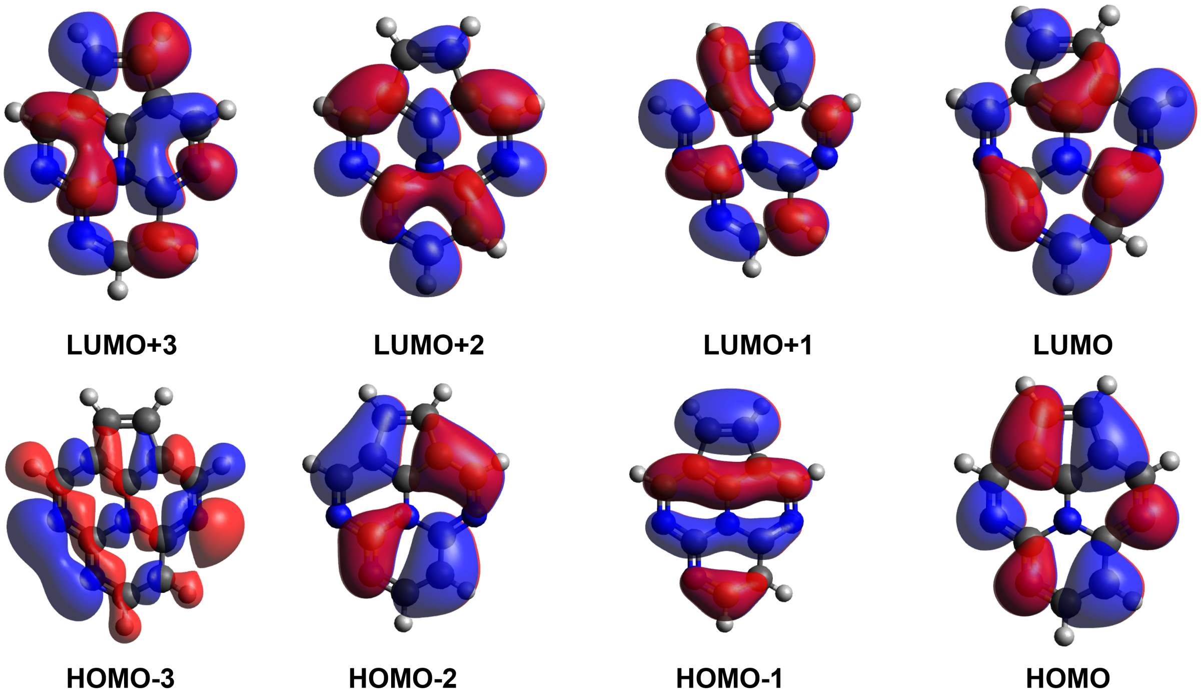

Fig. S35: HF orbitals used in CASSCF of known molecule 6. Isosurface value = 0.015.

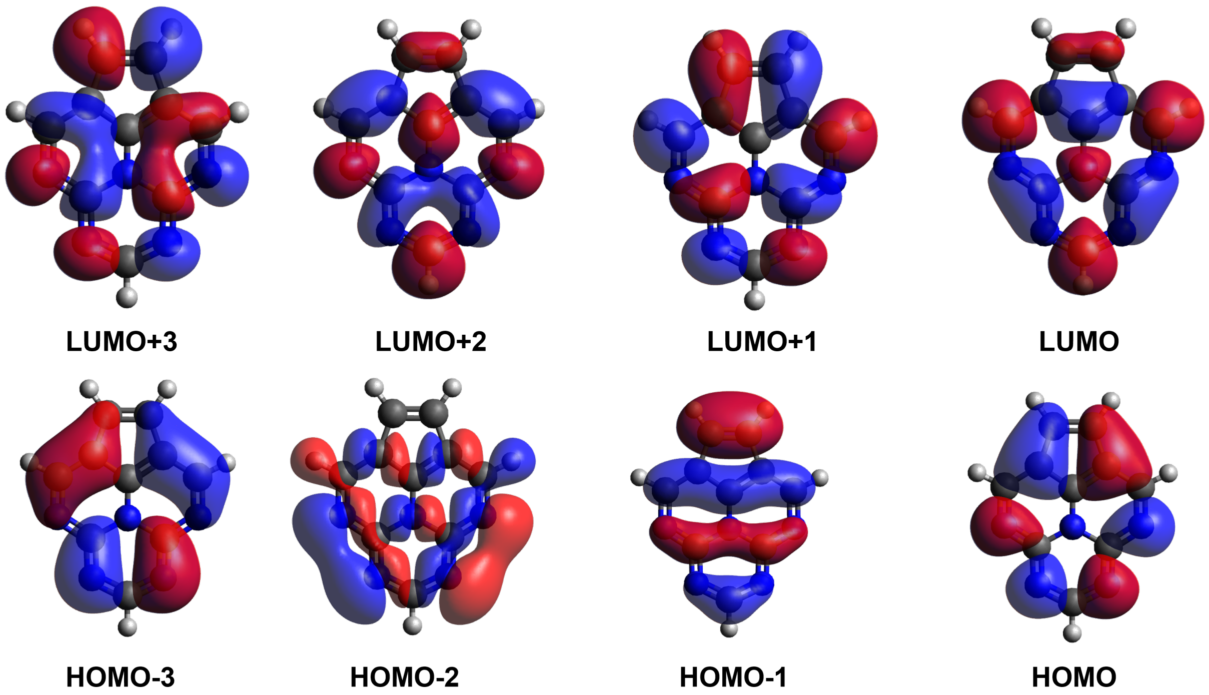

Fig. S36: HF orbitals used in CASSCF of known molecule 7. Isosurface value = 0.015.

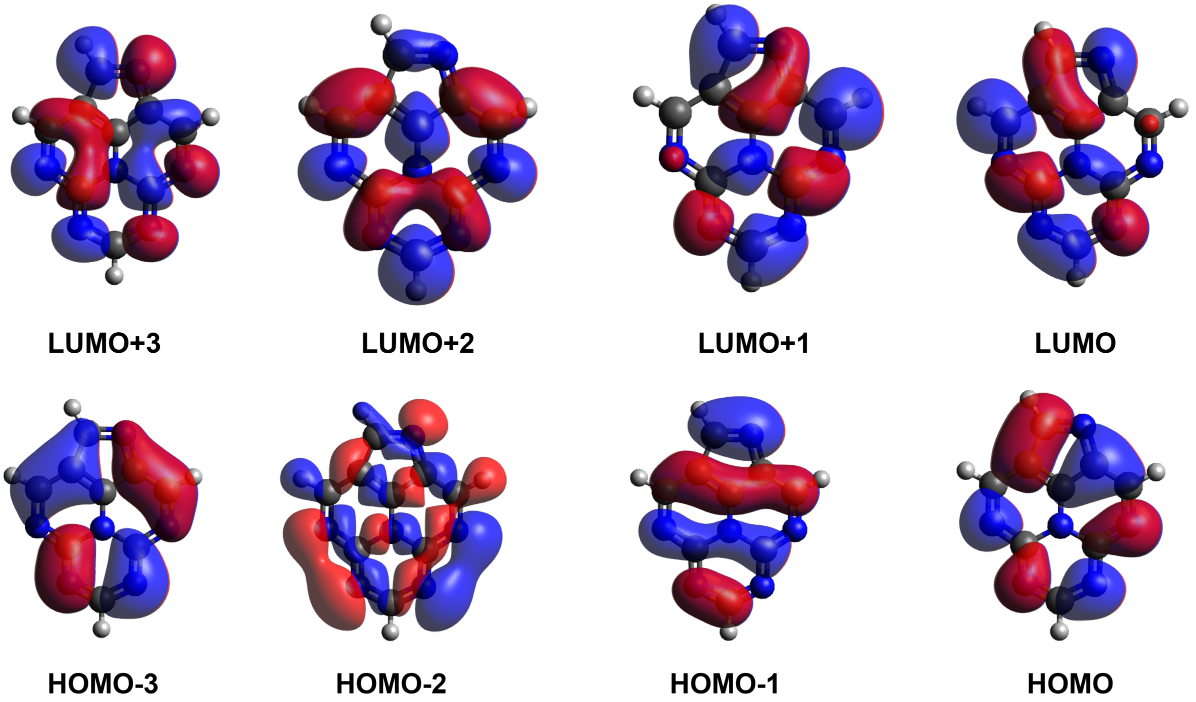

Fig. S37: HF orbitals used in CASSCF of known molecule 8. Isosurface value = 0.015.

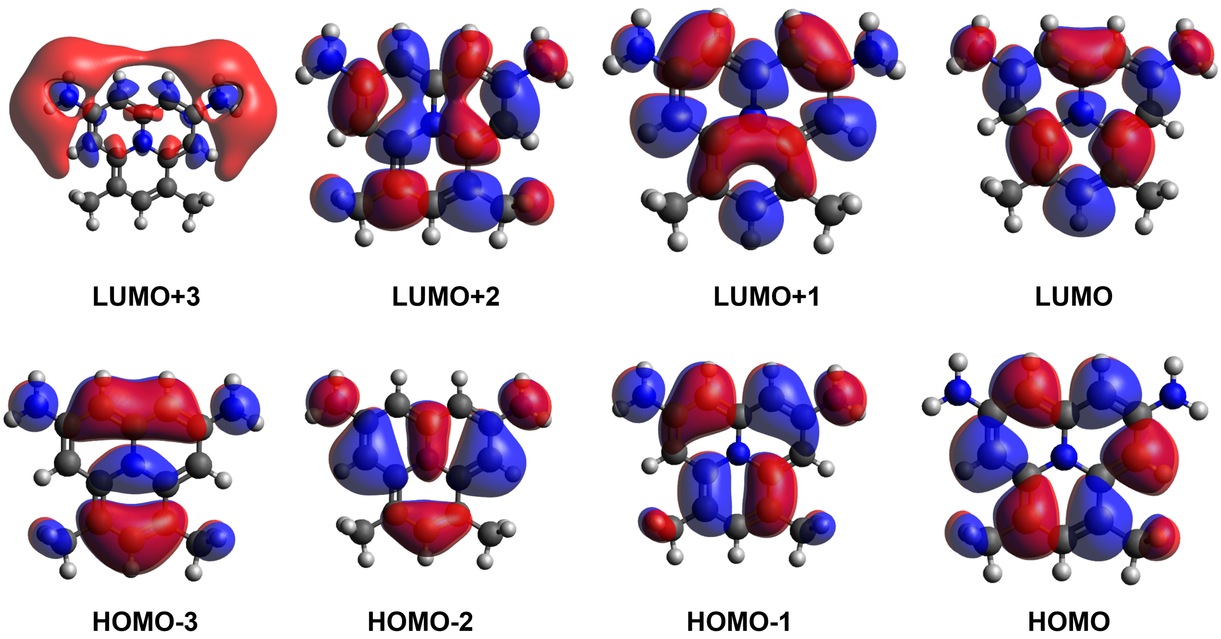

Fig. S38: HF orbitals used in CASSCF of known molecule 9. Isosurface value = 0.015.

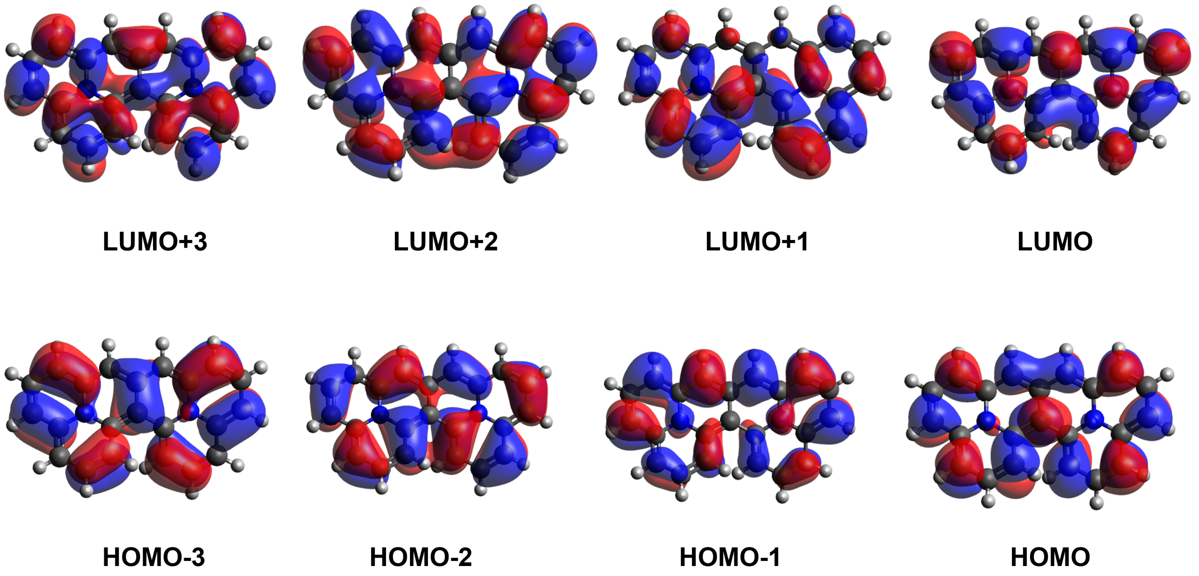

Fig. S39: HF orbitals used in CASSCF of known molecule 10. Isosurface value = 0.015.

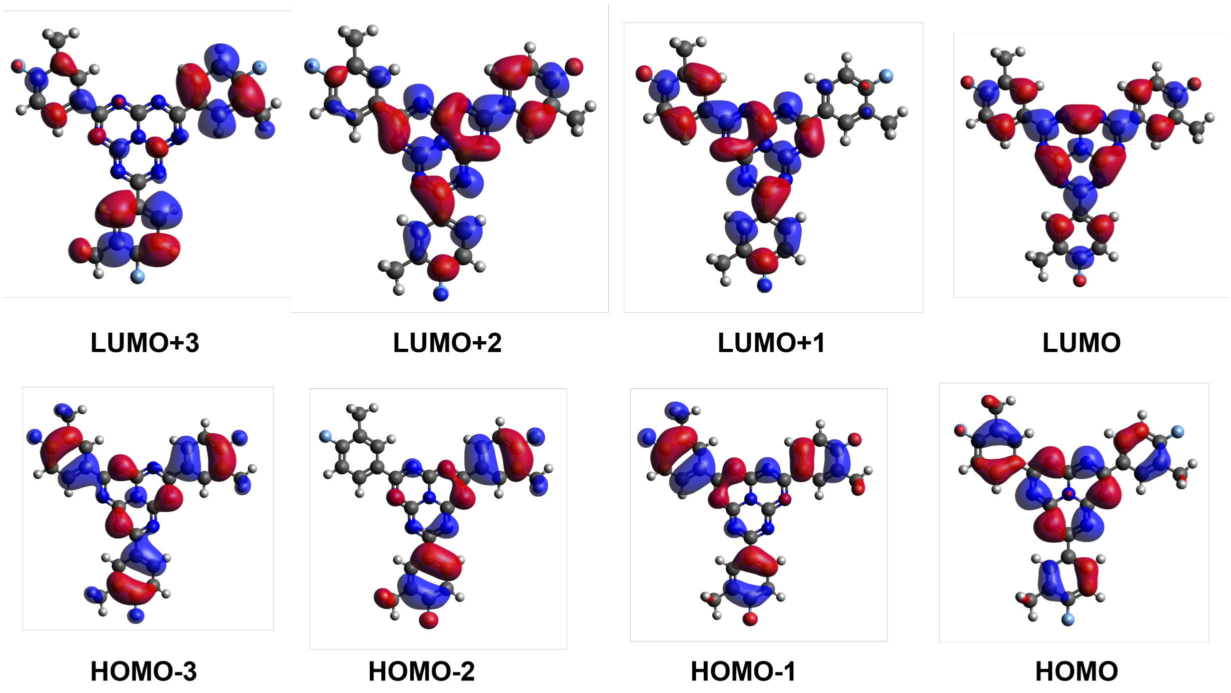

Fig. S40: HF orbitals used in CASSCF of known molecule 11. Isosurface value = 0.015.

S6 Comparison between geometries used for vertical and adiabatic evaluations of ΔE_{ST}

Table S2: RMSD between ground state (S_0) and S_1 and T_1 equilibrium geometries used in the calculation of vertical (see Table 2 in the main text) and adiabatic (see Table 3 in the main text) ΔE_{ST}.

Molecule	RMSD S_0-S_1 / Å	RMSD S_0-T_1 / Å	RMSD S_1-T_1 / Å
a	0.082	0.035	0.049
b	0.097	0.024	0.096
c	0.022	0.020	0.003
d	0.054	0.026	0.053
e	0.042	0.042	0.001
f	0.118	0.412	0.305
g	0.121	0.115	0.009
$g^{\dagger}$	0.035	0.035	0.003

†: RMSD computed from core atoms only.
